# Liver X receptors induce antiproliferative effects in basal‐like breast cancer

**DOI:** 10.1002/1878-0261.13476

**Published:** 2023-06-30

**Authors:** Mads Haugland Haugen, Hedda von der Lippe Gythfeldt, Eivind Valen Egeland, Lisa Svartdal Normann, Abhilash D. Pandya, Lise‐Lotte Vedin, Siri Juell, Ellen Tenstad, Geir Frode Øy, Alexandr Kristian, Elisabetta Marangoni, Therese Sørlie, Knut Steffensen, Gunhild Mari Mælandsmo, Olav Engebraaten

**Affiliations:** ^1^ Department of Tumor Biology Oslo University Hospital Oslo Norway; ^2^ Department of Cancer Genetics, Institute for Cancer Research Oslo University Hospital Norway; ^3^ Department of Oncology Oslo University Hospital Norway; ^4^ Insitute for Clinical Medicine University of Oslo Norway; ^5^ Department of Research and Innovation Vestre Viken Hospital Trust Drammen Norway; ^6^ Division of Clinical Chemistry, Department of Laboratory Medicine Karolinska Institutet Stockholm Sweden; ^7^ Translational Research Department, Institut Curie PSL Research University Paris France; ^8^ Department of Medical Biology, Faculty of Health Sciences The Arctic University of Norway‐University of Tromsø Norway

**Keywords:** basal‐like breast cancer, LXR, PDX, RPPA

## Abstract

Liver X receptors (LXRs) are nuclear transcription factors important in the regulation of cholesterol transport, and glucose and fatty acid metabolism. The antiproliferative role of LXRs has been studied in a variety of malignancies and may represent a therapeutic opportunity in cancers lacking targeted therapies, such as triple‐negative breast cancer. In this study, we investigated the impact of LXR agonists alone and in combination with carboplatin in preclinical models of breast cancer. *In vitro* experiments revealed a dose‐dependent decrease in tumor cell proliferation in estrogen receptor‐positive breast cancer cells, whereas LXR activation *in vivo* resulted in an increased growth inhibitory effect in a basal‐like breast cancer model (in combination with carboplatin). Functional proteomic analysis identified differences in protein expression between responding and nonresponding models related to Akt activity, cell‐cycle progression, and DNA repair. Furthermore, pathway analysis suggested that the LXR agonist in combination with carboplatin inhibits the activity of targets of E2F transcription factors and affects cholesterol homeostasis in basal‐like breast cancer.

## Graphical abstract

Activated liver X receptor (LXR) induces the expression of downstream target genes *ABCG1*, *SREBP1c*, and *FASN* and modulates Akt signaling. The combination of LXR agonist GW3965 and carboplatin reduces the proliferation of basal‐like breast cancer patient‐derived xenografts through reduced activity of targets of E2F transcription factors, reduced heregulin protein expression, and increased activity of cholesterol homeostasis.

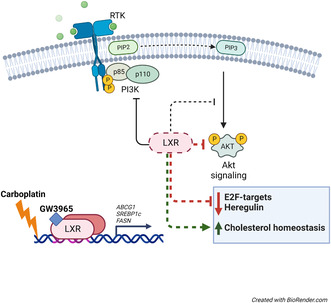

**Abbreviations**
*ABCG1*
: ATP‐binding cassette subfamily G member 1
CDK: cyclin‐dependent kinase
DNA: deoxyribonucleic acid
ER: estrogen receptor
FASN: fatty acid synthase
FC: fold change
FDR: false discovery rate
IPKB: ingenuity pathway knowledge base
LXR: liver X receptor
mRNA: messenger ribonucleic acid
mTOR: mammalian target of rapamycin
NR: nuclear receptor
PDX: patient‐derived xenograft
PI3K: phosphatidylinositol 3‐kinase
Rb: retinoblastoma
RPPA: reverse‐phase protein array
*SREBP1c*
: sterol regulatory element binding 1c
TNBC: triple‐negative breast cancer

## 1 Introduction

Identification of druggable targets and new treatment combinations is of importance for the development of new cancer therapies. Nuclear receptors (NRs), such as hormone receptors, are druggable targets of particular interest because they are activated by specific ligands [1]. They have been studied as prognostic and predictive factors in breast cancer and are well known as targets for endocrine therapy, improving the outcome for patients with luminal‐like tumors. Triple‐negative breast cancers (TNBCs), of which the majority are of the basal‐like subtype, are lacking such receptors. These malignancies have limited treatment opportunities, and the need for effective targeted therapies is particularly urgent.

Liver X receptors (LXRs), LXRα and LXRβ, encoded by the genes *NR1H3* and *NR1H2*, are members of the NR superfamily of ligand‐activated transcription factors. LXRα is highly expressed in prostate, breast, colon, pancreatic, esophageal, and liver tissues, whereas LXRβ is ubiquitously expressed [2]. LXRs regulate gene expression by binding to LXR response elements in the promotor region of responsive genes and mediate their biological effects through transcriptional regulation of their targets. Two such LXR‐responsive genes are *ABCG1* (ATP‐binding cassette subfamily G member 1) and *SREBP1c* (sterol regulatory element binding 1c). The LXR receptors are associated with metabolic functions including cholesterol homeostasis, fatty acid homeostasis, inflammation, and immunity [3, 4]. Due to their role in cell proliferation and tumor development, LXRs have been widely investigated as potential targets for prevention and treatment of several cancers, and considerable work has been carried out *in vivo* and *in vitro* to determine the role of LXRs in breast cancer [5, 6, 7]. Activation of LXRs by synthetic agonists has been shown to reduce proliferation in several breast cancer cell lines, and transcriptional profiling showed that the downregulated genes mostly participated in cell‐cycle regulation, DNA (deoxyribonucleic acid) replication, and other proliferation‐related processes [8].

The phosphatidylinositol 3‐kinase (PI3K)/Akt/mammalian target of rapamycin (mTOR) pathway plays an important role in regulating cell growth, proliferation, apoptosis, angiogenesis, and protein synthesis. Dysregulation of this pathway is one of the most frequent oncogenic aberrations in TNBC and most pronounced in the basal‐like subtype. Activation of the PI3K/Akt/mTOR pathway is primarily mediated at the protein level, and phosphorylation of Akt at serine 473 is necessary to fully activate the pathway [9, 10]. This pathway is also involved with regulation of cell metabolism and fatty acid synthesis [11], and induces *de novo* lipogenesis by increased activation and expression of SREBP1 and FASN (fatty acid synthase) [12]. LXR activation intercepts several intracellular signaling cascades and results in inactivation of the PI3K/Akt/mTOR pathway by dephosphorylation of phosphatidylinositol trisphosphate (PIP_3_) and Akt. This pathway might represent the most important target in the antiproliferative effects of LXR agonists [13].

GW3965, a synthetic LXR ligand that specifically binds and activates LXRs, can block the proliferation of estrogen receptor (ER)‐positive and ER‐negative breast cancer cells through downregulation of cell‐cycle‐ and growth‐associated genes, indicating that LXRs may function through both ER‐dependent and ER‐independent mechanisms. Most preclinical experiments rely on cell lines; however, these do not reflect the heterogeneity of breast tumors and such results must be interpreted with caution [5, 6, 8, 14]. Patient‐derived xenograft (PDX) models have, in contrast, been shown to better retain the cell differentiation, morphology and heterogeneous architecture of the original tumor. Thus, PDX models representing the luminal and basal‐like subtypes are well suited to identify mechanisms underlying the LXR effects in hormone‐dependent and hormone‐independent breast cancers. Such models will simultaneously provide the opportunity to study LXR‐induced growth inhibition *in vivo* [13, 15].

Combination therapy involving several drugs is increasingly used as an approach to combat treatment resistance in cancer [3]. Carboplatin is a chemotherapeutic drug known to be efficient in TNBC [16]. In this study, we explored the effects of combining the LXR agonist GW3965 with carboplatin and investigated growth inhibition and target gene expression in tumors from three PDX models. Reverse‐phase protein array (RPPA) was used to study the effect of LXR activation and identified important targets on the cell proteome and in cancer‐relevant signaling pathways.

## 2 Materials and methods

### 2.1 Cell culture

The human basal‐like ER‐negative cell line MDA‐MB‐468 (CVCL_0419), was cultured in Dulbecco's modified Eagle's medium (Sigma‐Aldrich, St. Louis, MO, USA) containing 5.6 mm glucose, 1 mm sodium pyruvate, 4 mm l‐glutamine, 25 mm *N*‐2‐hydroxyethylpiperazine‐*N*′‐2‐ethanesulfonic acid, and 8% FBS (Sigma‐Aldrich). The human ER‐positive ductal breast carcinoma cell line T47D (CVCL_0553) (subtype luminal A) and the human ER‐negative cell line MDA‐MB‐231 (CVCL_0062) (claudin low) were cultured in Roswell Park Memorial Institute medium (RPMI; Sigma‐Aldrich) containing 11 mm glucose, 2 mm l‐glutamine and 8% FBS. All cell lines were cultivated in 25‐ and 75‐cm^2^ flasks (Corning Inc., Corning, NY, USA) at 37 °C, 5% CO_2_ in a humidified incubator and were routinely tested for mycoplasma (Venor^®^GeM; Minerva Biolabs, Skillman, NJ, USA). All cell lines were acquired from ATCC (Manassas, VA, USA) and routinely authenticated using the 13 core CODIS short tandem repeats loci plus Penta E, Penta D and the gender‐determining locus amelogenin.

### 2.2 Proliferation assay

A total of 6–8 × 10^3^ cells were plated in 96‐well plates (Falcon, Corning Inc.), incubated for 24 h and treated as indicated with increasing concentrations (1.25, 2.5, 5, and 10 μm) of GW3965 or vehicle (dimethylsulfoxide [DMSO]), followed by incubation for 7 days.

The absolute number of viable cells (proliferation) and percentage viable cells of the total cell population (viability) was analyzed by the CellTiter 96^®^ AQ_ueous_ One Solution Cell Proliferation Assay (Promega, Madison, WI, USA) according to the manufacturer's protocol.

### 2.3 Animal experiments

Patient‐derived breast cancer xenograft (PDX) models were established at the Institute for Cancer Research, Oslo University Hospital, Norway (MAS98.12, MAS98.06) [13] and Institute Curie, France (HBCx39) [15]. These tumor models originated from the implantation of biopsy tissues from primary mammary carcinomas into foxn1nu nude mice as previously described. The study methodologies conformed to the standards set by the Declaration of Helsinki. The MAS 98.06 and MAS98.12 tumor tissue were obtained from patients undergoing routine surgery for the removal of breast cancer in 1998. When the tissues were established as continuously growing patient‐derived xenografts, it was not possible to retrieve a written informed consent. In accordance with the Declaration of Helsinki, the use of breast cancer tissue for research purposes was therefore approved by the Regional Committee for Medical Research Ethics in South‐Eastern Norway (approval no. S‐07398a). The HBCx39 model was originally established at Institute Curie in Paris in 2009 and transferred to Oslo University Hospital in 2012 for use in this institution. A written informed consent was obtained from the patient before the use of this tissue for research purposes. All models were routinely bilaterally transplanted as 1‐ to 3‐mm^3^ pieces into nude mice (age 6–8 weeks) under inhalation anesthesia (Sevoflurane, Baxter, IL, USA). A small incision was made above the sternum, and tumor pieces were inserted under the skin in the area around the thoracic mammary glands. All mice were bred at the Department of Comparative Medicine, Oslo University Hospital‐Norwegian Radium Hospital. They were kept under pathogen‐free conditions and had free access to food and water. All procedures involving animals and experimental protocols were approved by the Norwegian Animal Research Authority and conducted in accordance with the guidelines of the Federation of European Laboratory Animal Science Association (FOTS no. 10 296).

A total of 75 female athymic nude *Foxn1* ^*nu*^ mice were included in this study (20 mice in the experiment with the luminal‐like MAS98.06 and 36 and 19 mice in the experiments with the basal‐like MAS98.12 and HBCx39 xenografts, respectively). For the two combination treatment experiments with the basal‐like PDX models, the mice were divided into four treatment groups: control, carboplatin (Hospira Nordic AB, Stockholm Sweden), GW3965 (Sigma‐Aldrich/Merck, KGaA, Darmstadt, Germany) and carboplatin/GW3965 (combination). Each group contained 7–11 mice, in total 12–16 tumor samples in each group, with an average tumor volume distribution of approximately 50 mm^3^ on day 0.

Carboplatin, 100 mg·kg^−1^ diluted in 0.9% saline, was administered in the lower right quadrant of the abdomen of the mouse with an intraperitoneal injection once a week for 3 weeks, secured by a gentle grip. The carboplatin dose was selected based on literature search, and a separate small dose‐finding study. Due to signs of toxicity in the first experiment, the carboplatin dose was reduced to 50 mg·kg^−1^ in the follow‐up studies. GW3965, 40 mg·kg^−1^ [17], diluted in 0.5% methylcellulose (Sigma‐Aldrich/Merck, KGaA), was given 7 days a week by gavage for the duration of the experiment. During administration of GW3965, the mice were secured by hand so that the head and neck of the mice were fixated. A 18 G stainless steel feeding tube with a slight curve was then inserted into the mouth and led down the esophagus of the mouse, and GW3965 was slowly administered into the stomach of the mouse. All treatments were administered in a volume equal to 10 μL·g^−1^ body weight. The combination group received both carboplatin and GW3965, as described above, whereas the control group was left untreated. The mice were observed after the procedures and resumed normal activity without signs of distress related to the treatment procedures. The body weight of the mice was measured daily, and the tumor diameters were measured twice weekly using digital calipers. Tumor volume was estimated by the formula 0.5 × length × width^2^. The mice were checked daily for signs of deteriorated health related to the treatments, with endpoints including body weight loss > 10–15%, weakened health and tumor volume > 1500 mm^3^. At the end of the experiments, the animals were sacrificed by cervical dislocation and tumor tissue was harvested from each of the xenograft models, immediately frozen in liquid nitrogen at −80 °C, and stored under cryogenic conditions until analysis.

### 2.4 RNA extraction and microarray hybridization of xenograft tissue

The total RNA from snap‐frozen xenograft tissue samples was isolated using TRIzol reagent (Invitrogen, Carlsbad, CA, USA) and measured using NanoDrop (NanoDrop Technologies, Wilmington, DE, USA). A total of 100–125 ng RNA were amplified and labeled following the Agilent Low Input Quick Amplification Labeling Kit (Agilent, Santa Clara, CA, USA) protocol for one‐color, microarray‐based, gene expression analysis. Hybridization was performed according to the manufacturer's protocol and scanned using an Agilent Technologies Microarray Scanner (G2505C; Agilent).

### 2.5 Quantitative polymerase chain reaction

The total RNA was isolated using EZNA Total RNA Kit I (Omega Bio‐Tek, Norcross, GA, USA) according to the manufacturer's protocol. Complementary DNA synthesis was performed using SuperScript II RT (Invitrogen) with random hexamers. Messenger (m)RNA expression was quantified on an Applied Biosystems QuantStudio 12K Flex (Applied Biosystems, Waltham, MA, USA) instrument using the SYBR green technology. Data were analyzed using the comparative *CT* method, with 18S RNA as an internal control.

### 2.6 Proteomic analyses by RPPA and simple western immunoassay

Eight samples from the therapy studies in the HBCx39 PDX model (two from each treatment group) and 12 samples from the MAS98.12 PDX model (three from each treatment group) were prepared for RPPA analysis. Snap‐frozen tumor samples were mechanically grinded into powder while continuously being kept frozen using liquid nitrogen. Protein lysates were extracted from the tumor powder using T‐PER Tissue Protein Extraction Reagent (Thermo Fisher Scientific, Waltham, MA, USA) containing phosphatase inhibitor, PhosSTOP, and protease inhibitor cOmplete Tablets (Roche, Indianapolis, IN, USA) for 1 h, before sonication and centrifugation of the samples. The supernatants were collected, and protein concentrations were determined using a Pierce BCA Protein Assay Kit (Thermo Fisher Scientific), according to the provider's protocol, and measured on a Victor X Plate Reader (PerkinElmer, Waltham, MA, USA).

The RPPA profiling was performed at the RPPA core facility of MD Anderson Cancer Center (Houston, TX, USA) as previously described [18]. Briefly, serial diluted protein lysates were arrayed onto nitrocellulose‐coated slides (Grace Bio‐labs, Bend, OR, USA) using an Aushon 2470 Arrayer (Aushon BioSystems, Billerica, MA, USA), including the spots corresponding to positive and negative controls prepared from mixed‐cell lysates and dilution buffer, respectively. Each slide was probed with a validated primary antibody plus a biotin‐conjugated secondary antibody.

Each dilution curve was fitted with a logistic model (‘Supercurve Fitting’), developed by the Department of Bioinformatics and Computational Biology at MD Anderson Cancer Center [19]. All the data were normalized for protein loading and transformed to log_2_ values. Differences between PDX models and treatment groups were presented as volcano plots, with colored points representing differently expressed proteins using cutoffs for fold‐change (FC) > 0.5 and *P*‐values < 0.05 (unadjusted).

Proteins of interest in MAS98.12 or in HBCx39 PDX were measured by simple western immunoassay, using a Peggy Sue™ instrument (ProteinSimple, San Jose, CA, USA). The lysate concentration was adjusted to 1.0 μg·μL^−1^. Protein separation was performed using a 12–230 kDa separation master kit (SM‐S001; ProteinSimple) in accordance with the manufacturer's protocol. Primary antibody incubation time was adjusted to 60 min, while all the other settings were kept on default. The compass software (ProteinSimple, version 5.0.1) was used to program the experimental setup and to collect and analyze the data. The following antibodies were used: Heregulin (2573; Cell Signaling Technology, Danvers, MA, USA) 1 : 50, anti‐β‐actin (A5316; Sigma, St. Louis, MO, USA) 1 : 100, anti‐pAKT‐S473 (9271; Cell Signaling Technology), 1 : 25, anti‐pAKT‐T308 (9275; Cell Signaling Technology), 1 : 25, anti‐pS6‐S235/236 (4858; Cell Signaling Technology), 1 : 50, anti‐ p70 S6 kinase‐T389 (9205; Cell Signaling Technology), 1 : 50, anti‐pGSK3β‐S9 (9336; Cell Signaling Technology), 1 : 50, anti‐pmTOR‐S2448 (5536; Cell Signaling Technology), 1 : 50. Other related reagents (PS‐ST01EZ) as well as anti‐rabbit (DM‐001) and anti‐mouse detection modules (DM‐002) were acquired from ProteinSimple (San Jose, CA, USA).

### 2.7 Pathway analysis

The r package GSVA (v1.44.5) [20] was used to assess variation in signaling pathways and functions within the RPPA samples. Single sample scores were calculated with log‐transformed normalized RPPA data and the Hallmark database (retrieved with r package msigdbr (v7.5.1)) used as input for the *gsva* function. To reduce false positives, a minimum of 5 genes had to be represented in a pathway for the pathway to be included for scoring. For comparing the two models, all samples were used as input to calculate the score values, while changes in treated samples were estimated with MAS98.12 samples only.

Score values were used for generating heatmaps with the r package pheatmap (v1.0.12) by mean centering gsva output per pathway. Unsupervised clustering was performed with clustering method complete and distance Euclidean.

Finally, differential expression analysis in pathway activation was assessed using the limma package (v 3.52.4) [21] with gsva score values as input. Volcano plots were generated with the output (*P*‐value and log_2_FC) from the differential expression analysis.

### 2.8 Statistical analysis

Statistical analysis was performed in r version 3.3.1 [22]. The paired Student's *t*‐test was used to determine the differences in proliferation between the groups treated with different GW3965 concentrations in the *in vitro* experiments. All measurable tumors at the end of the experiments were included in the statistical analysis of the *in vivo* experiments. The association between treatment groups in the tumor models was assessed using Wilcoxon's rank‐sum test. The significance of differences in protein expression between groups was calculated using a two‐sample Student's *t*‐test, leaving the *P*‐values unadjusted due to the restricted number of samples. All statistical tests were two‐sided, and a *P*‐value < 0.05 was considered to be significant.

## 3 Results

### 3.1 LXR inhibits proliferation in human breast cancer cell lines

**Fig. 1:**
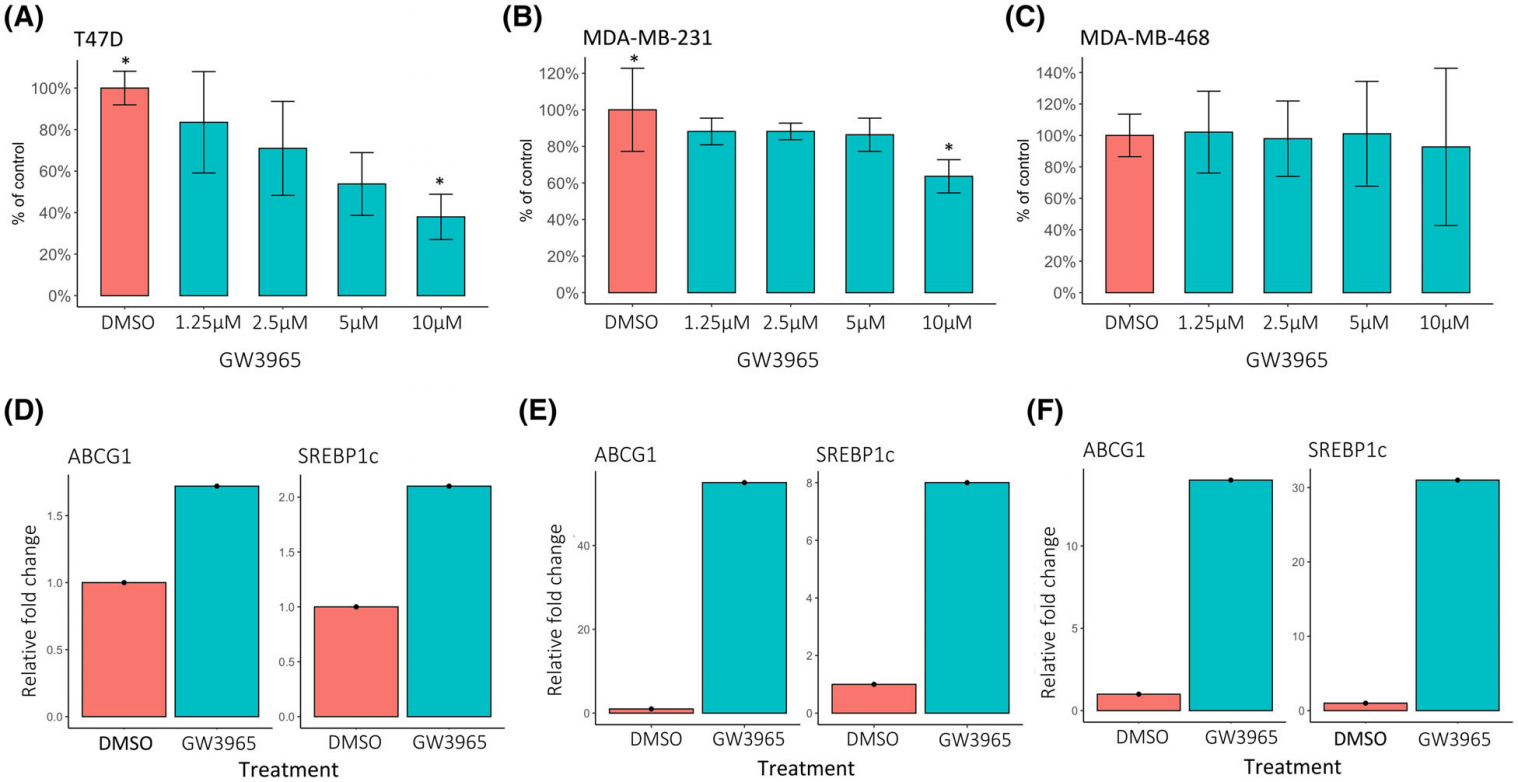
Effects of LXR activation in luminal and basal‐like breast cancer cell lines. A colorimetric cell proliferation assay (MTS) was used to detect the inhibitory effect of increasing concentrations of the LXR agonist GW3965 after 7 days in (A) T47D (*n* = 3) (luminal cell line), (B) MDA‐MB‐231 (*n* = 3) and (C) MDA‐MB‐468 (*n* = 3) (both basal‐like cell lines). All proliferation assays are presented as percentage of vehicle (DMSO) and error bars represent SEM. *Student's *t*‐test *P* < 0.05. (D–F) Relative mRNA expression of target genes (*ABCG1* and *SREBP1c*) on treatment with 10 μm GW3965 (*n* = 2) compared with DMSO (*n* = 1) in T47D, MDA‐MB‐231 and MDA‐MB‐468, respectively. The relative expression of DMSO treatment was set to 1.0 and data presented as mean.

Response to activation by the synthetic LXR agonist GW3965 was investigated in the ER‐positive cell line T47D and the two triple‐negative cell lines MDA‐MB‐231 and MDA‐MB‐468. A dose‐dependent decrease in cell proliferation on treatment was detected, resulting in a significant growth inhibitory effect in two of the cell lines, T47D and MDA‐MB‐231 (Student's *t*‐test *P* < 0.05 at GW3965 10 and 5 μm versus vehicle [DMSO] in T47D, and GW3965 10 μm versus DMSO in MDA‐MB‐231; Fig. 1A–C). GW3965 induced the expression of the LXR target genes *ABCG1* and *SREBP1c* in all three cell lines (Fig. 1D–F).

### 3.2 LXR activation by synthetic ligand reduces tumor growth in combination with carboplatin in a basal‐like PDX

The influence of LXR activation was further investigated *in vivo* using PDX models. In a luminal‐like model (MAS98.06), treatment with GW3965 did not inhibit tumor growth, but influenced the expression of the LXR target genes *ABCG1* and *SREBP1c* (Fig. S1). In the basal‐like model (MAS98.12), a dose–response experiment resulted in reduction of tumor growth compared with control at a dose of 40 mg·kg^−1^ with borderline significance (Wilcoxon's *P* = 0.059). Furthermore, a significant induction in expression of the target gene *ABCG1* was observed (Student's *t*‐test *P* < 0.05) (data not shown). GW3965 at 40 mg·kg^−1^ was therefore selected as the optimal dose for further experiments.

**Fig. 2:**
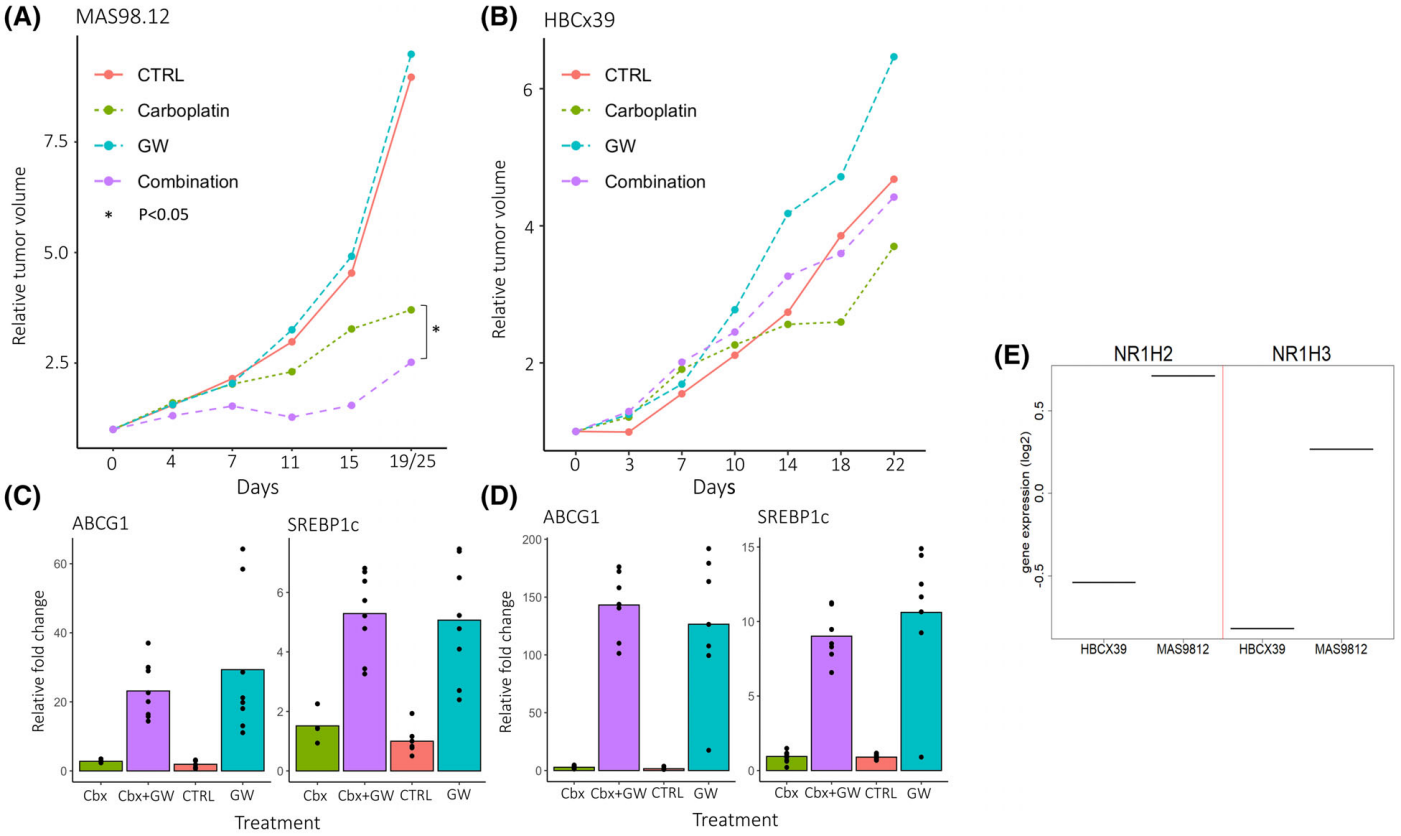
LXR activation in patient‐derived xenograft models of basal‐like breast cancer. Growth curves showing the effect of the LXR agonist GW3965 (GW) in combination with carboplatin, presented as a median relative tumor volume in (A) MAS98.12 and (B) HBCx39. The addition of GW3965 to carboplatin resulted in a significant reduction in tumor growth in MAS98.12 *P < 0.05 (Wilcoxon's *P* = 0.022), but not in HBCx39 (Wilcoxon's *P* = 0.089). Number of tumors in each group MAS98.12: CTRL (*n* = 19), GW (*n* = 16), Carboplatin (*n* = 12), Combination (*n* = 13) HBCx39: CTRL (*n* = 10), GW (*n* = 7), Carboplatin (*n* = 11), Combination (*n* = 5). (C, D) LXR activation by GW3965 induced the expression of target genes in both tumor models. Number of tumors in each group MAS98.12: CTRL (*n* = 7), GW (*n* = 8), Carboplatin (*n* = 4), Combination (*n* = 8) HBCx39: CTRL (*n* = 10), GW (*n* = 6), Carboplatin (*n* = 12), Combination (*n* = 7). (E) Gene expression analysis detected a higher expression of LXRα encoded by *NR1H3* and LXRβ encoded by *NR1H2* in MAS98.12 compared with HBCx39 (data derived from RNA expression array analysis of one tumor from each tumor model). CTRL, control; Cbx, carboplatin; GW, GW3965; Combination/Cbx + GW, carboplatin and GW3965.

Combination therapy with GW3965 and carboplatin in the MAS98.12 model significantly reduced the relative tumor volume compared with carboplatin alone (Wilcoxon's *P* = 0.022; Fig. 2A). Single‐agent therapy with the optimal dose of GW3965 had no growth inhibitory effect in this experiment. In the HBCx39 basal‐like breast cancer model, the addition of GW3965 to carboplatin did not significantly reduce tumor growth (Wilcoxon's *P* = 0.089; Fig. 2B). Importantly, treatment with GW3965, alone or in combination, consistently induced the expression of the LXR target genes *ABCG1* and *SREBP1c*, independent of the effect on tumor growth (Fig. 2C,D).

The two LXR receptors, LXRα and LXRβ, are encoded by the genes *NR1H3* and *NR1H2*. These genes were relatively higher expressed in MAS98.12 compared with HBCx39, which could explain the observed differences in LXR activation between the two models (Fig. 2E).

### 3.3 Functional proteomics suggests targets for the antiproliferative effect of LXR

The mechanisms underlying the antiproliferative effect of LXR activation are largely unknown. We used proteomic data from RPPA to explore altered protein expression patterns between the tumor models and pathways associated with LXR activation.

**Fig. 3:**
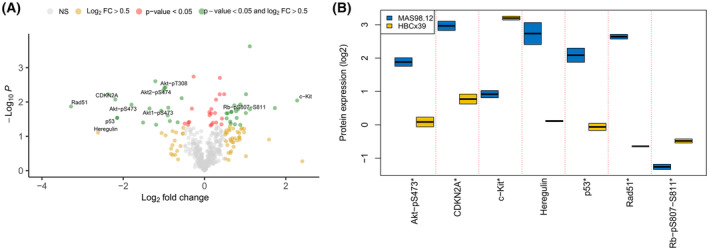
Protein expression by RPPAs in basal‐like patient‐derived xenograft (PDX) models HBCx39 compared with MAS98.12 before treatment. (A) Volcano plot demonstrating magnitude of fold‐change protein expression (log_2_(FC); *x*‐axis) and statistical significance of this change (Student's *t*‐test *P*‐value; −log_10_, *y*‐axis) in HBCx39 (*n* = 2) versus MAS98.12 (*n* = 2) untreated controls. Colored points represent differently expressed proteins in which green points have a significant *P‐*value (< 0.05) and |log_2_(FC)| > 0.5, red points have a significant *P*‐value and |log_2_(FC)| < 0.5, whereas the yellow and gray points have a nonsignificant (NS) *P*‐value and a |log_2_(FC)| > 0.5 or < 0.5, respectively. (B) Boxplot demonstrating selected differently expressed proteins between the MAS98.12 and HBCx39 PDX models from same samples. *Student's *t*‐test unadjusted *P* < 0.05.

Overall differences in protein expression between the untreated PDX models (MAS98.12 and HBCx39) are illustrated in Fig. 3A. Of the 386 proteins analyzed, 39 proteins met the criteria of an (absolute) |FC| > 0.5 and an unadjusted *P*‐value < 0.05 (Table S1). Selected proteins were involved in cell‐cycle transition and DNA repair, with higher expression of Akt‐pS473, CDKN2A (p16‐INK4a), Heregulin, p53 and Rad51, and lower expression of the receptor tyrosine kinase c‐Kit and Rb‐pS807‐S811 in MAS98.12 compared with HBCx39 (Fig. 3B). However, the difference in protein expression did not reach statistical significance after correction for multiple testing (false discovery rate [FDR]‐adjusted *P*‐values). In a separate analysis, the signal from both pAkt‐S473 and pAkt‐T308 and the downstream signal pGSK‐3β‐S9 were significantly higher the MAS98.12 model than in the HBCx39 model, while small or no difference was found between the models for other Akt downstream signals like pmTOR‐S2448, p70 S6 kinase‐T389, and pS6‐S235/236 (Fig. S2).

**Fig. 4:**
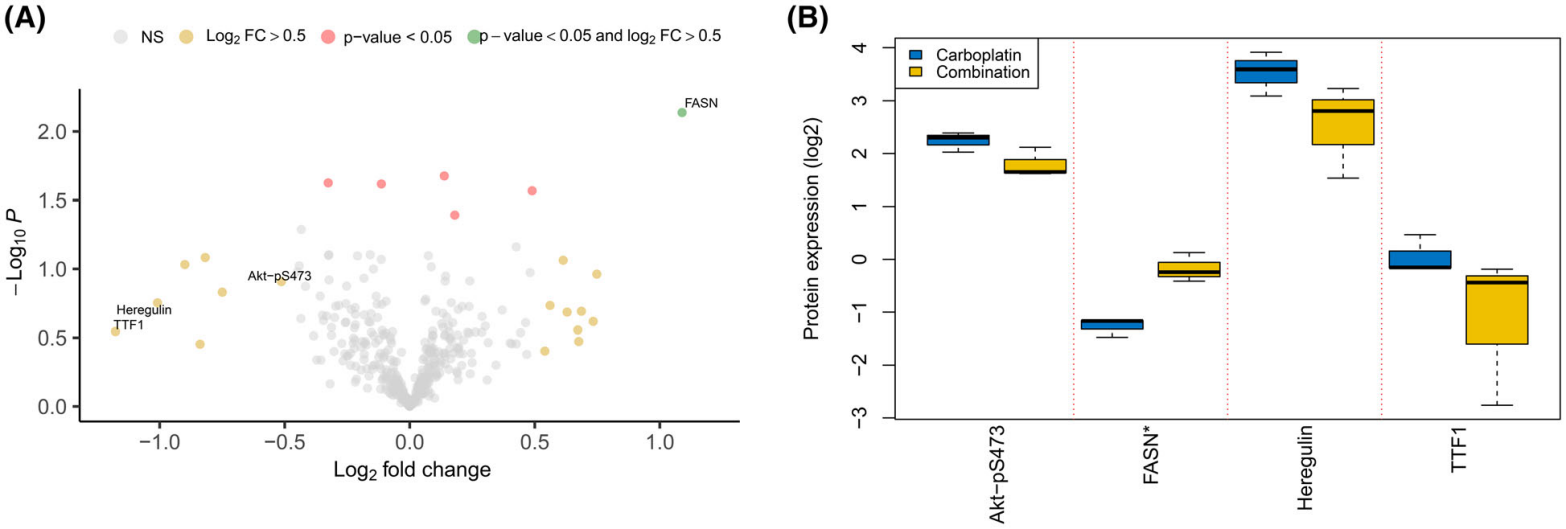
Protein expression by RPPAs comparing carboplatin and GW3965 combination versus carboplatin alone‐treated MAS98.12. (A) Volcano plot demonstrating magnitude of fold‐change protein expression (log_2_(FC); *x*‐axis) and statistical significance of this change (Student's *t*‐test *P*‐value −log_10_; *y*‐axis) between samples treated with GW3965 and carboplatin combination (*n* = 3) versus carboplatin alone (*n* = 3) in MAS98.12. Colored points represent differently expressed proteins in which green points have a significant *P*‐value (< 0.05) and |log_2_(FC)| > 0.5, red points have a significant *P*‐value and |log_2_(FC)| < 0.5, whereas the yellow and gray points have a nonsignificant (NS) *P*‐value and |log_2_(FC)| > 0.5 or < 0.5, respectively. (B) Boxplot demonstrating four of the differently expressed proteins between the same combination‐ and carboplatin‐treated samples *Student's *t*‐test unadjusted *P* < 0.05.

Tumor samples were collected at the end of treatment from control animals and animals treated with GW3965 alone and in combination with carboplatin. LXR activation with GW3965 resulted in a significant reduction in tumor proliferation when combined with carboplatin in the MAS98.12 model, and changes in protein expression between the different treatment arms are therefore of particular interest. The expression differences induced by LXR activation in combination with carboplatin compared with carboplatin mono treatment were modest (Table S2), with only the known LXR activation target FASN reaching the cutoff |FC| > 0.5 and *P* < 0.05 (Fig. 4A). However, other proteins like Heregulin and TTF1 being involved in cell proliferation, as well as Akt‐pS473, had decreased expression in the combination treatment compared with carboplatin alone (Fig. 4B). Using an orthogonal protein detection method, lower expression of Heregulin was observed with the combination treatment while no difference was observed between the treatment groups for the pGSK3β‐S9 and pmTOR‐S2448 Akt downstream targets (Fig. S3). In HBCx39, few additional effects on protein expression as measured by RPPA were observed when adding GW3965 to carboplatin in HBCx39, and regulation of the known LXR target FASN was not significant and very modest compared with the MAS98.12 model. Similarly, no regulation of the Heregulin nor TTF1 was observed, while expression of Akt‐pS473 was higher in the carboplatin treatment compared with combination, as opposed to the MAS98.12 model. In HBCx39 only, the difference in expression of two proteins remained significant between tumors treated with carboplatin or combination treatment (CDH1 and TIGAR, data not shown). Hence, LXR activation modestly alter protein expression in both models, but resulted in different effects on both protein targets and tumor growth.

### 3.4 Identification of biological pathways and networks involved in LXR activation

**Fig. 5:**
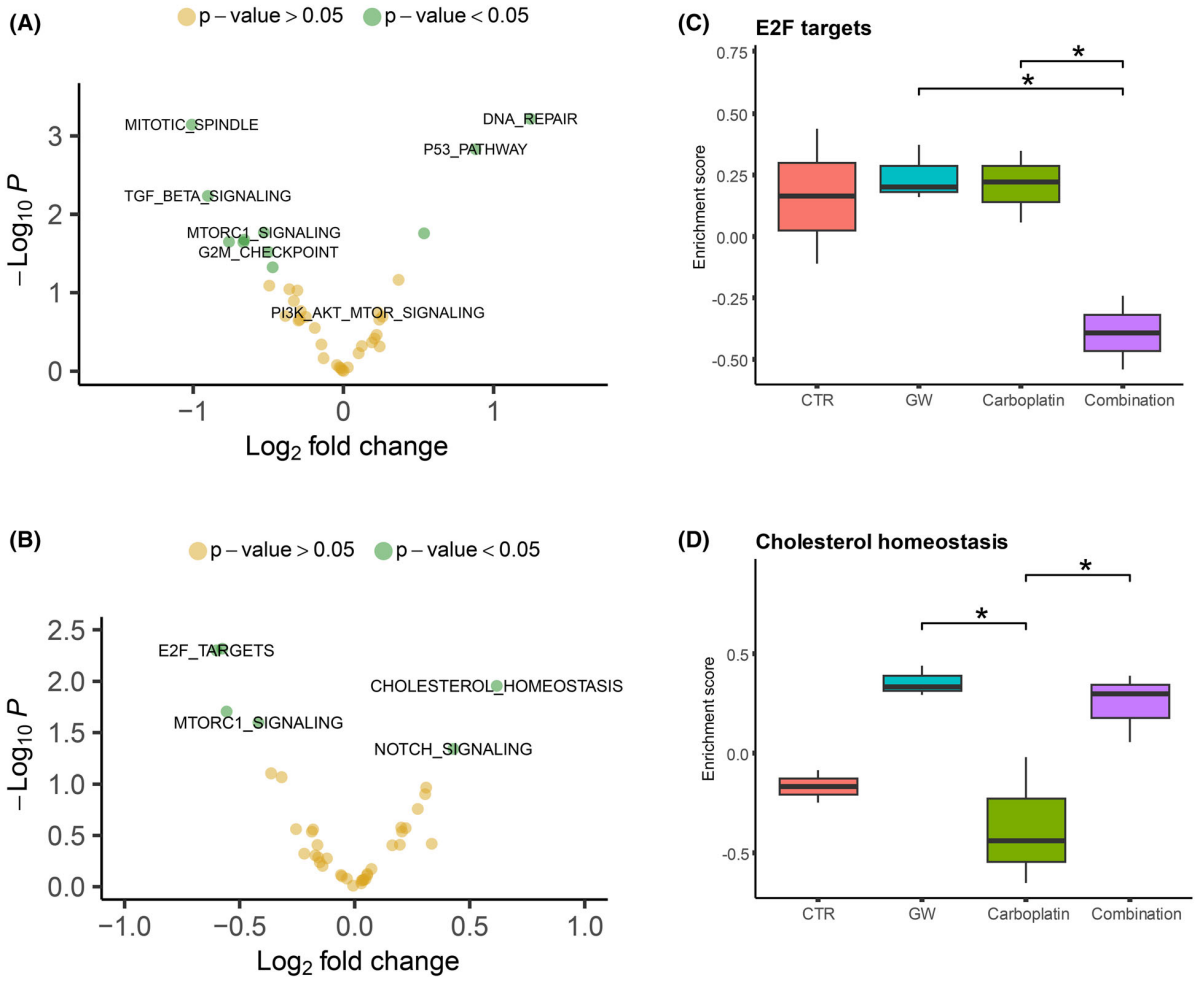
Pathway activation in basal‐like breast cancer xenografts models before and after treatment with LXR agonist GW3965. Gene Set Variation Analysis (GSVA) was used to generate single sample scores for gene signatures in the Hallmark database base on the RPPA proteomic data. Differential expression analysis was then performed with the r package limma, and changes in pathway enrichment between the groups of comparison are displayed as volcano plot with magnitude of fold‐change pathway enrichment score (log_2_FC; *x*‐axis) and statistical significance of this change (pairwise *t*‐test unadjusted *P*‐value −log_10_; *y*‐axis). *P*‐values < 0.05 was considered significant (green). (A) Pathway enrichment comparing the two basal‐like models MAS98.12 (*n* = 2) and HBCx39 (*n* = 2) at baseline. (B) Pathway enrichment comparing the carboplatin and GW3965 combination (*n* = 3) versus carboplatin alone (*n* = 3) treated MAS98.12 tumor samples. Several Hallmark signatures showed significant changes in enrichment between combination and carboplatin, and single sample scores were used to assess distribution across all treatment groups in MAS98.12. (C) A distinct drop in enrichment of E2F genes, associated with proliferating cancer cells, was observed in the combination group (*n* = 3), but not in the other treatment groups (control; *n* = 2, GW and carboplatin; *n* = 3). (D) Cholesterol homeostasis‐associated genes showed increased enrichment in both groups treated with the LXR agonist GW3965 (*n* = 3), but not with carboplatin (*n* = 3) alone or in the control group (*n* = 2). *Pairwise *t*‐test adjusted (Bonferroni) *P* < 0.05. CTR, control; GW, GW3965; Combination, carboplatin and GW3965.

To further explore biological pathways associated with LXR activation, we assessed how gene signatures from the Hallmark database changed between the treatment groups by employing GSVA on the RPPA data. First, the differences between the two untreated xenograft models were investigated by GSVA to generate single scores, followed by differential pathway expression (Fig. 5A, Fig. S4). We identified 11 pathways with significant change in enrichment between the models. Among pathways with higher enrichment in MAS98.12 were DNA repair and the p53 pathway the most significant. Overall PI3K/AKT/mTOR‐signaling was slightly higher in MAS98.12, while genes downstream of mTORC1 were higher in HBCx39, suggesting a slight difference in PI3K/AKT/mTOR activation between the models. In addition, G2M checkpoint and mitotic spindle showed higher enrichment in the HBCx39.

Furthermore, we sought to identify the most relevant biological processes involved in the mechanism behind the LXR effect by analyzing RPPA data from MAS98.12 tumors treated with the LXR agonist in combination with carboplatin versus carboplatin alone (Fig. 5B, Fig. S5). Analyses revealed that E2F targets were among the pathways with lower enrichment in the combination group. Comparing across all treatments, this group was significantly lower than control, GW3965, and carboplatin (Fig. 5C). Interestingly, we observed an increase in cholesterol homeostasis in the combination treatment. Assessing the relative level of enrichment across all treatments, this increase was observed in both the treatment groups receiving GW3965 alone as well as in the combination (Fig. 5D).

## 4 Discussion

Modulators of NRs serve as potential cancer drugs and may potentiate the effect of conventional treatment. A number of therapies have recently been proven effective in the treatment of TNBC, such as immunotherapy with atezolizumab or pembrolizumab in combination with chemotherapy, PARP inhibitors or targeted therapy with antibody‐drug conjugates such as trastuzumab deruxtecan or sacituzumab govitecan. However, malignancies such as TNBC may often be treatment resistant, or lack some of these new targeted treatment opportunities, and are then potential candidates for such treatment. LXRs are NRs that have been shown to promote cancer cell death through the effect of their ligands in preclinical studies of various cancers, partly due to reduced activity of the PI3K/Akt/mTOR pathway [9, 23, 24]. Our *in vitro* experiments confirmed previous results, demonstrating a pronounced antiproliferative effect of the LXR agonist in luminal breast tumors [6, 8]. The *in vivo* experiments revealed new insights into the mechanism behind LXR activation used alone and in combination with chemotherapy, in basal‐like breast tumors. Combination therapy resulted in an enhanced growth inhibitory effect and might represent a promising treatment option in selected cases of breast cancer.

**Fig. 6:**
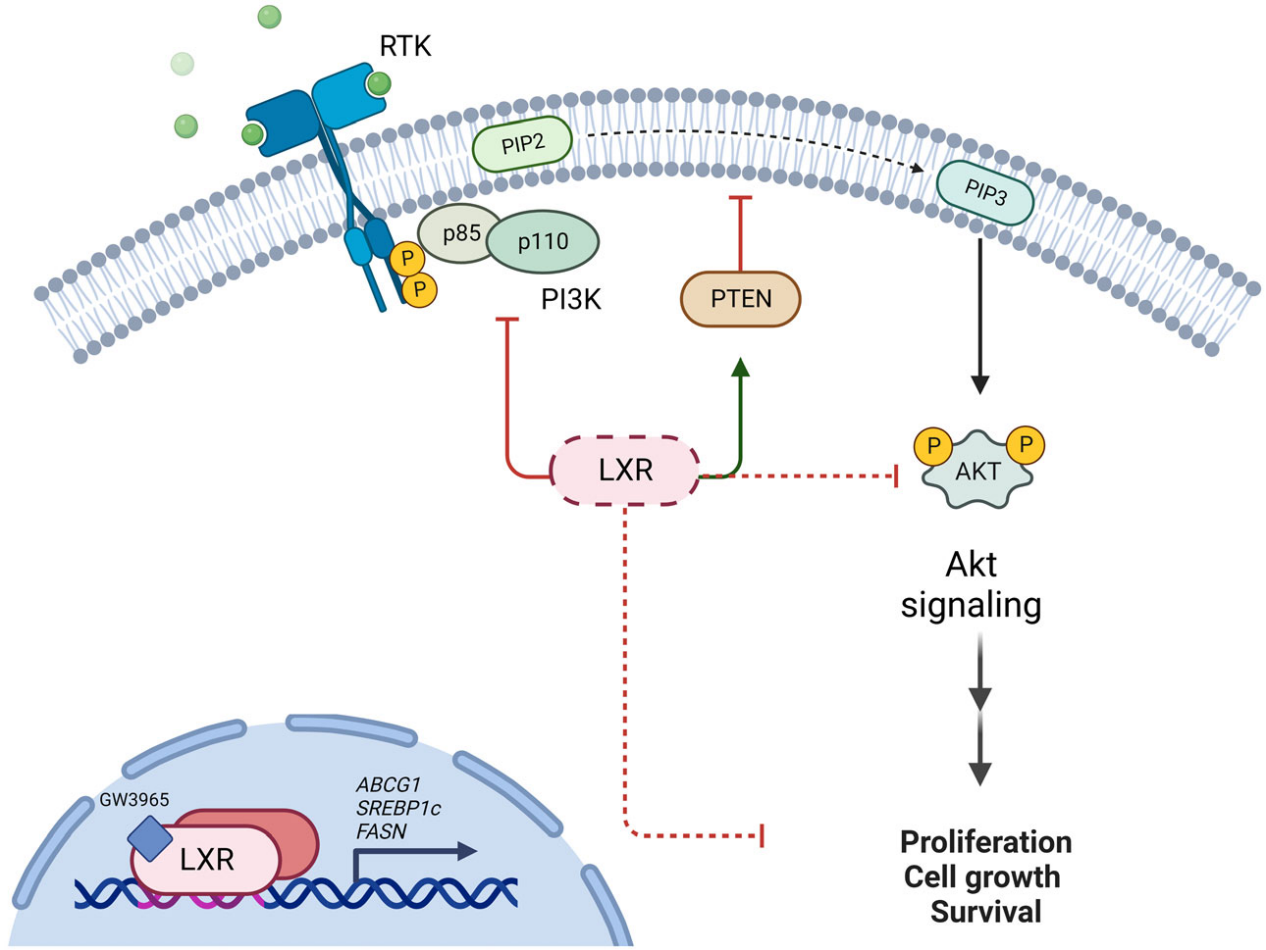
Schematic illustration of downstream effects of LXR activation. GW3965 primarily activates LXR dimerization (with the corresponding Retinoid X receptor), dissociation from corepressor, and recruitment with coactivators. Activated LXR induces the expression of downstream target genes like *ABCG1*, *SREBP1c*, and *FASN*. Studies have also shown LXR‐mediated decrease in PI3K‐subunit phosphorylation and corresponding increase of PTEN activation, as well as modulation of AKT signaling by LXR agonist activation. Altogether, this results in reduced proliferation, growth, and survival of cells (Created with BioRender.com).

Liver X receptors and their ligands function through various mechanisms (Fig. 6). Their role in endocrine‐related cancers suggests a potential for application as a new therapeutic option in specific disease subtypes that resist conventional therapies or currently do not have adequate therapeutic options, such as refractory TNBC.

Previous studies examining differences in LXR signaling across breast cancer subtypes not only reported antiproliferative effect in ER‐positive cell lines but also enhanced transcription activity from LXR target genes in ER‐negative cell lines [25].

Results from our *in vitro* experiments indicated the most pronounced effect of LXR activation in the ER‐positive cell lines, but this was not translated *in vivo*, suggesting that hormone‐independent mechanisms are involved in mediating the LXR effect. Using single‐cell RNA‐sequencing data, LXR activity has been shown to be upregulated in TNBCs, and that these tumor cells produce endogenous LXR ligands that activate downstream signaling in immune cells such as macrophages and cytotoxic T cells [26]. Cholesterol metabolism is frequently altered in cancer, associated with increased proliferation and angiogenesis [27]. In this study, we observed an increased enrichment for cholesterol homeostasis upon treatment with GW3965 alone, or in combination with carboplatin, in the MAS98.12 model (Fig. 5D). This is a previously observed function of the LXR activation [4]. *In vitro* experiments performed by Hutchinson *et al*. [28] uncovered increased expression of LXR regulatory factors in ER‐negative disease, indicating that ER‐negative tumors are particularly sensitive to increased cholesterol. In our monotherapy experiments, the basal‐like PDX model showed a growth inhibitory response to LXR ligands of borderline significance not apparent in the luminal PDX. The effect was also reflected by the induction of target genes, most pronounced in the basal‐like model.

Liver X receptor activation by GW3965 resulted in diverse effects on tumor proliferation in the two basal‐like PDX models, suggesting that the different expression levels of LXR receptors and affiliated pathways could be involved in the antiproliferative mechanisms. Protein expression analysis was performed [29] and detected proteins related to cell‐cycle progression, DNA repair, fatty acid synthesis, and the PI3K/Akt/mTOR pathway, which differed between the PDX models and between treatment groups.

Triple‐negative breast cancer is a heterogeneous disease, and alterations in the components of the cell‐cycle machinery have frequently been reported [30, 31]. The PI3K/Akt/mTOR pathway plays a critical role in the control of cell growth, proliferation, migration, and metabolism, and aberrations in this pathway are distinctive features of TNBC [10]. When comparing the untreated MAS98.12 and HbCx39 models, we found that Akt‐pS473, Akt‐pT308, Akt1‐pS473, and Akt2‐pS474 all had an increased expression in MAS98.12 (Fig. 3, Fig. S2), which may explain the effect of LXR activation on tumor growth observed in this model. We further observe an effect of this increased Akt signaling in MAS98.12 on downstream activation of pGSK3β‐S9, but not on pmTor‐S2448, pS6‐S235/236 or p70 S6 kinase‐T389 (Fig. S2). In line with previously published data, we demonstrated that LXR activation was associated (although not significant) with reduced phosphorylation of Akt‐pS473 (Fig. 4), leading to impaired activity of this pathway [9, 32]. However, as no reduction in expression of pGSK3b‐S9 nor pmTOR‐S2448 was observed, signaling leading to impaired tumor growth must be through other downstream effectors (Fig. S3). FASN, a known LXR activation target, was significantly upregulated in the combination‐ vs carboplatin‐treated MAS98.12 (Fig. 4). Additionally, a decreased expression of the proliferation‐associated proteins Heregulin [33]and TTF1 [34] was observed in the combination treatment (Fig. 4, Fig. S3). Heregulin has a number of other tumor promoting effects as well like VEGF secretion and angiogenesis [35], and the downregulation could explain the effect of the combination treatment on tumor growth in MAS98.12. An association between LXR and Heregulin [36] in mice, as well as Heregulin and FASN expression, has previously been observed in Luminal B‐like BC [37]. However, the systems biology mechanistically linking all these factors together needs further elucidation. Pathway analysis revealed significantly reduced activity/enrichment of E2F targets in the combination arm (Fig. 5C). This reflects the observed reduction in proliferation/growth in the combination arm, as the signature contains genes encoding cell‐cycle‐related targets of the E2F transcription factors. One study in ER+ BC demonstrated how high expression of Hallmark E2F targets is associated with aggressiveness and MKI67‐expression [38], thus, reduction of such signatures in tumors with better response to treatment aligns with the observed reduction in proliferation/growth in the combination group. Interestingly, in a recent study LXR was reported to control DNA repair and apoptosis, and could thus interact with the effect of carboplatin [39]. Such an interaction may influence the reduction of tumor growth in the combination group, and not with carboplatin alone in our treatment‐sensitive model.

Liver X receptor activation by GW3965 influenced the expression of proteins involved in cell‐cycle‐associated pathways, representing a potential strategy in the treatment of TNBC. However, in our study the measurement of the molecular factors is done after a protracted treatment period at the end of the experiment. At this time, the growth of the tumors, although inhibited in the combination‐treated tumors compared with the other treatment groups, demonstrates a pattern of regrowth. It can therefore not be excluded that some activated proteins detected are linked to the emergence of treatment resistance. This is highly relevant, as resistance inevitably occurs in such a treatment setting, and strategies to combat such treatment resistance are much needed. Treatment with LXR ligand has been shown to block cell‐cycle progression and phosphorylation of retinoblastoma protein (Rb) [6]. In this study, we did observe a significant difference in expression of Rb‐pS807‐S811 between the MAS98.12 HbCx39 models; however, no significant effect in expression levels was observed when treated with GW3965. The Rb protein is the main target in the CDK4/6 pathway and is a key protein in the control of cell growth. A large fraction of TNBC cases has functional Rb (reported in 80% of basal‐like tumors), which are predominantly TNBC with the potential for response to CDK 4/6‐targeted therapy. These inhibitors are well established in treatment of advanced hormone receptor‐positive breast cancer, with a significant improvement in disease‐free survival [40, 41, 42, 43]. Due to synergistic interactions between CDK 4/6 and PI3K inhibitors, there is a preclinical rationale for the combined targeting of these two pathways to block tumor growth in TNBC [31]. LXR targeting in combination with a CDK4/6 inhibitor might represent a therapeutic option in selected TNBCs.

Liver X receptor agonists have been combined with other antitumor agents with promising activity in preclinical studies of melanoma (vemurafenib, dacarbazine [DTIC], anti‐CTLA‐4 antibody) and pancreatic cancer (gemcitabine) [24, 44]. In addition, the first phase I clinical trial investigating the oral LXR agonist RGX‐104, in combination with immunotherapy and chemotherapy in advanced solid tumors and lymphoma, is ongoing [45].

Platinum agents demonstrate antitumor effects in TNBC, with an improved response rate in both the neoadjuvant and the metastatic setting, most pronounced in patients harboring a mutation in the *BRCA1/2* genes [46, 47, 48, 49]. In our *in vivo* experiments, combination therapy with carboplatin and the LXR agonist resulted in a statistically significant reduction in tumor cell proliferation in one of the basal‐like PDXs (MAS98.12), but not in the other (HBCx39). *RAD51* is a crucial factor in DNA repair, representing a surrogate marker of resistance to DNA‐damaging agents such as carboplatin [50]. The observed higher expression of this protein in MAS98.12 compared with HBCx39 may indicate a potential node of interaction for the observed response to combination therapy seen in these models, despite the similar effect of carboplatin monotherapy. Importantly, the effect on known LXR target genes was present in both models, despite the difference in LXR receptor expression.

## 5 Conclusions

Liver X receptor ligands target gene networks and pathways of importance for cell growth and proliferation, and most of the existing research in this field is preclinical. Our results demonstrate the effect of LXR‐targeted therapy in combination with a relevant commonly used therapy regimen in breast cancer and point to specific pathways involved in treatment response. This adds to the molecular knowledge of potential therapies that might be active in TNBC and may contribute to the development of new specific treatment regimens for patients with such tumors.

## Conflict of interest

The authors declare no conflict of interest.

## Author contributions

HvdLG, MHH, and OE drafted the manuscript. HvdLG was responsible for the animal experiments, acquired, and analyzed data from the *in vitro* and *in vivo* experiments. MHH was responsible for the analyses of proteomic data. EVE was responsible for analyses of proteomic data and involved in revision of the manuscript. LN acquired and analyzed proteomic data and was involved in the drafting of the manuscript. ADP was responsible for the simple western immunoassay analysis. GFØ and AK were responsible for data collection and animal experiments. L‐LV analyzed gene expression data. SJ and ET were responsible for the *in vitro* experiments. GMM, L‐LV, KS, EM, TS, and OE coordinated the animal models, designed and coordinated the study, and were involved in interpretation of the data and drafting of the manuscript. All authors contributed to the revision, editing, and the final version of the manuscript.

### Peer review

The peer review history for this article is available at https://www.webofscience.com/api/gateway/wos/peer-review/10.1002/1878-0261.13476.

## Supporting information


**Fig. S1.** Tumor growth curves and effect on target genes on the luminal MAS98.06 breast cancer xenograft treated with the Liver X receptor agonist GW 3965. (a) Growth curve showing the effect of GW3965 (40 mg/kg) compared with CTRL, presented as relative tumor volume in the luminal MAS98.06 breast cancer xenograft. (b) Liver X receptor activation by GW3965 induced the expression of target genes ABCG1 and SREBP1c in the luminal MAS98.06 breast cancer xenograft (Ctrl n = 13, GW3965 n = 18). Ctrl: control *** significance (Student's t‐test unadjusted *P* < 0.05).

**Fig. S2.** Protein expression by simple western immunoassay in breast cancer xenograft models MAS98.12 and HBCx39. Average fold protein expression (normalized to β‐actin) of pAKT‐S473, pAKT‐T308, pS6‐S235/236, pGSK3β‐S9, p70 S6 kinase‐T389, and pmTOR‐S2448 as measured by simple western immunoassay for MAS98.12 (n = 2) or HBCx39 (n = 2). pAKT expression in HBCx39 was not detected and thereby could not be quantified. Data are shown as mean with error bars representing SD. * unpaired parametric t‐test *P* < 0.05.

**Fig. S3.** Protein expression by simple western immunoassay in breast cancer xenograft model MAS98.12 treated with Liver X receptor GW3965 and carboplatin. Average fold protein expression (normalized to β‐actin) of Heregulin, pGSK3β‐S9, and pmTOR‐S2448 as measured by simple western immunoassay for control (n = 3), GW (n = 3), carboplatin (n = 2) and combination (n = 3) treatment groups in MAS98.12 tumors. Data are shown as mean with error bars representing SD. Statistical significance tested by unpaired parametric t‐test. GW: Liver X receptor GW3965.

**Fig. S4.** Heatmap of all Hallmark gene signature scores from Gene Set Variation Analysis (GSVA) in HBCx39 and MAS98.12 control tumors. Hallmark gene signature scores from Gene Set Variation Analysis (GSVA) in HBCx39 (n = 2) and MAS98.12 (n = 2) control tumors with unsupervised clustering of breast cancer xenograft models in heatmap. Scale indicates mean‐centered single sample scores across each pathway. Columns were unsupervised clustered with method complete and distance Euclidean.

**Fig. S5.** Heatmap of all Hallmark gene signature scores from Gene Set Variation Analysis (GSVA) in MAS98.12 carboplatin‐ and combination‐treated tumors. Hallmark gene signature scores from Gene Set Variation Analysis (GSVA) in MAS98.12 carboplatin (n = 3) and combination (n = 3) treated tumors with unsupervised clustering of treatment groups in heatmap. Scale indicates mean‐centered single sample scores across each pathway. Columns were unsupervised clustered with method complete and distance Euclidean.

**Table S1.** Protein expression by reverse‐phase protein array analysis in MAS98.12 and HBCx39 breast cancer xenograft tumors. Difference in expression of 386 proteins in HBCx39 (n = 2) compared with MAS98.12 (n = 2) untreated breast cancer xenograft tumors by reverse‐phase protein array analysis. Significance by Student's t‐test unadjusted and FDR adjusted p‐value.

**Table S2.** Protein expression by reverse‐phase protein array analysis in MAS98.12 carboplatin‐ and combination‐treated cancer xenograft tumors. Difference in expression of 386 proteins in the combination (n = 3) compared with carboplatin (n = 3) treated MAS98.12 breast cancer xenograft tumors. Significance by Student's t‐test unadjusted and FDR adjusted p‐value.

## Data Availability

Data generated during this study are included in this published article and its additional files. All data are available and will be collaboratively shared upon reasonable request.
