# Supplementary figures and images for: Liver X receptors induce antiproliferative effects in basal‐like breast cancer
Source: Mol Oncol. 2023 Jun 30;17(10):2041–55. doi: 10.1002/1878-0261.13476 (PMC10552888; doi:10.1002/1878-0261.13476)

Supplementary Figure S1

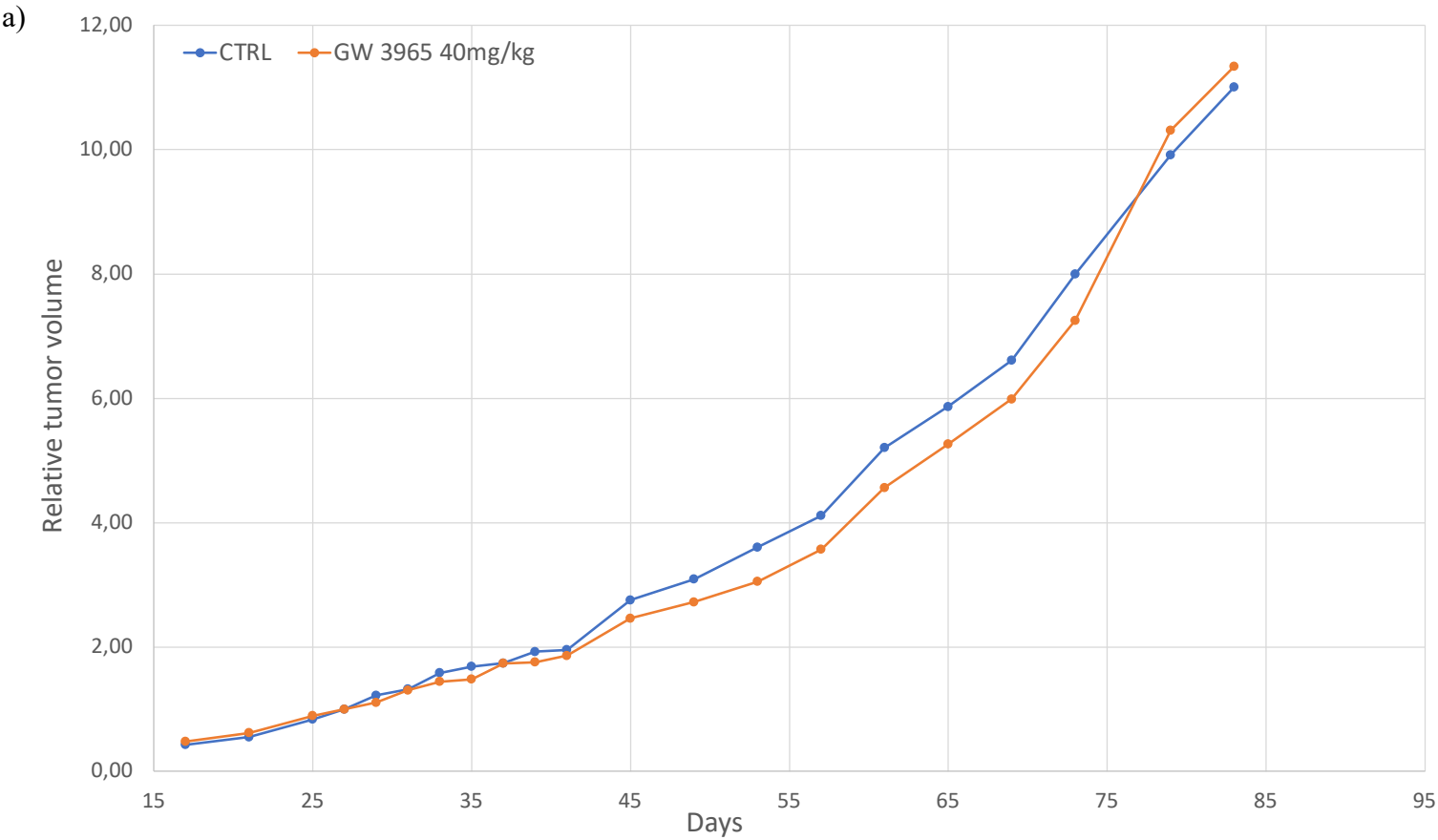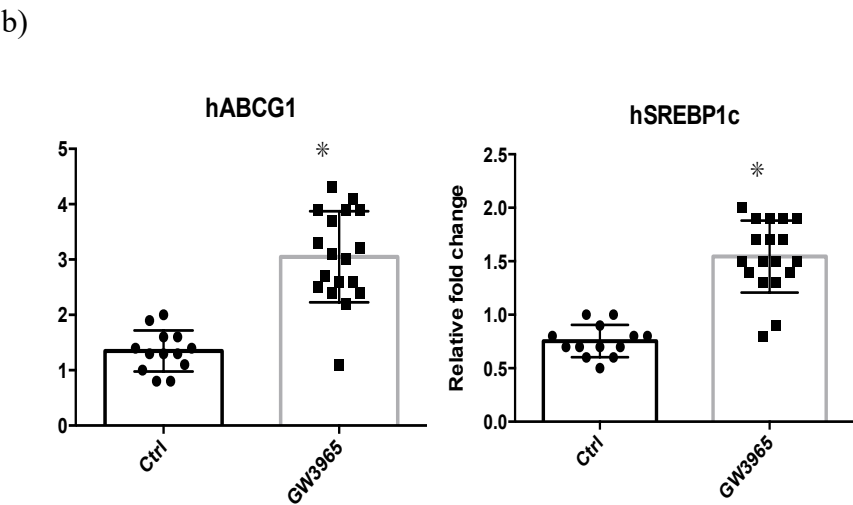

Supplement: Supplementary file 1 — Fig. S1. Tumor growth curves and effect on target genes on the luminal MAS98.06 breast cancer xenograft treated with the Liver X receptor agonist GW 3965. (a) Growth curve showing the effect of GW3965 (40 mg/kg) compared with CTRL, presented as relative tumor volume in the luminal MAS98.06 breast cancer xenograft. (b) Liver X receptor activation by GW3965 induced the expression of target genes ABCG1 and SREBP1c in the luminal MAS98.06 breast cancer xenograft (Ctrl n = 13, GW3965 n = 18). Ctrl: control *** significance (Student's t‐test unadjusted P < 0.05). [file MOL2-17-2041-s004.pdf]

Figure S2

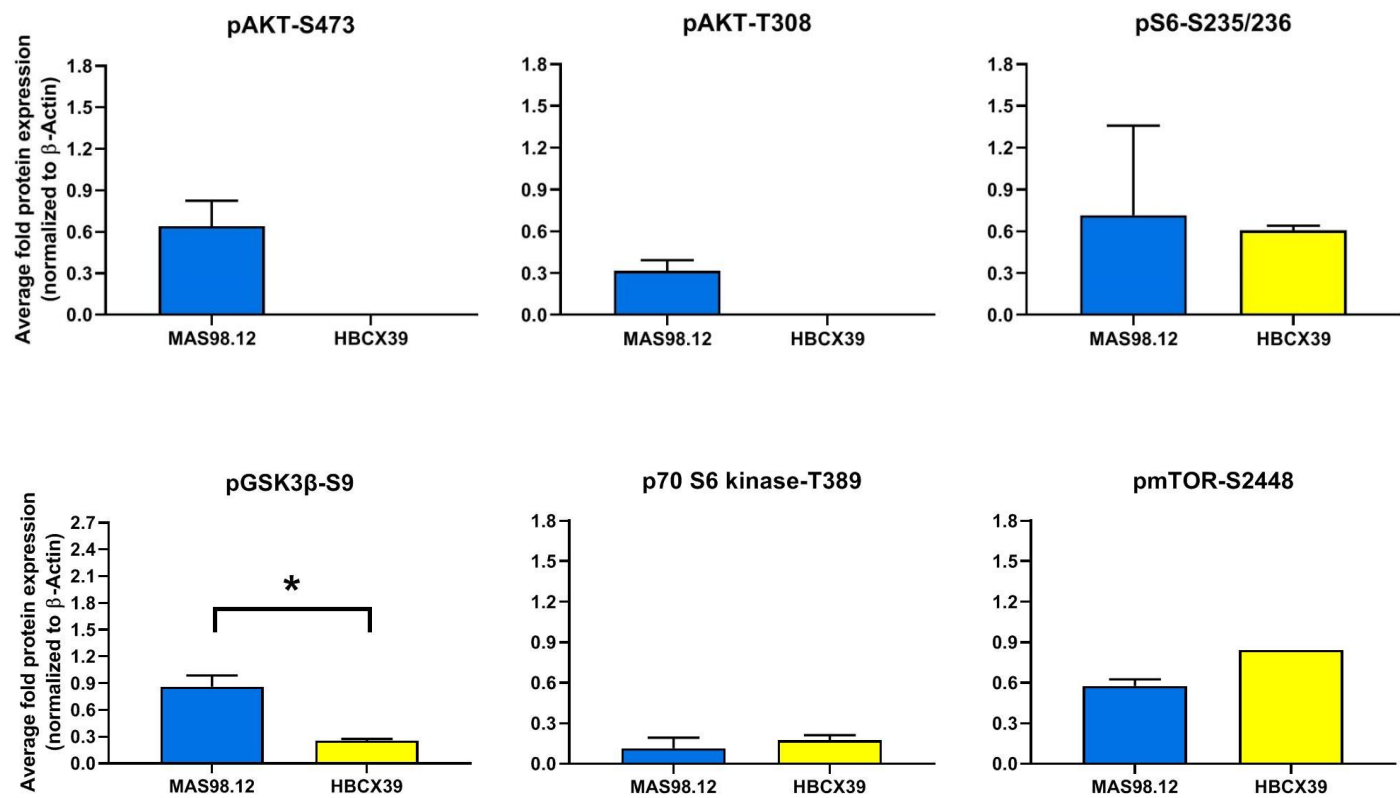

Supplement: Supplementary file 2 — Fig. S2. Protein expression by simple western immunoassay in breast cancer xenograft models MAS98.12 and HBCx39. Average fold protein expression (normalized to β‐actin) of pAKT‐S473, pAKT‐T308, pS6‐S235/236, pGSK3β‐S9, p70 S6 kinase‐T389, and pmTOR‐S2448 as measured by simple western immunoassay for MAS98.12 (n = 2) or HBCx39 (n = 2). pAKT expression in HBCx39 was not detected and thereby could not be quantified. Data are shown as mean with error bars representing SD. * unpaired parametric t‐test P < 0.05. [file MOL2-17-2041-s006.pdf]

Supplementary figure S3

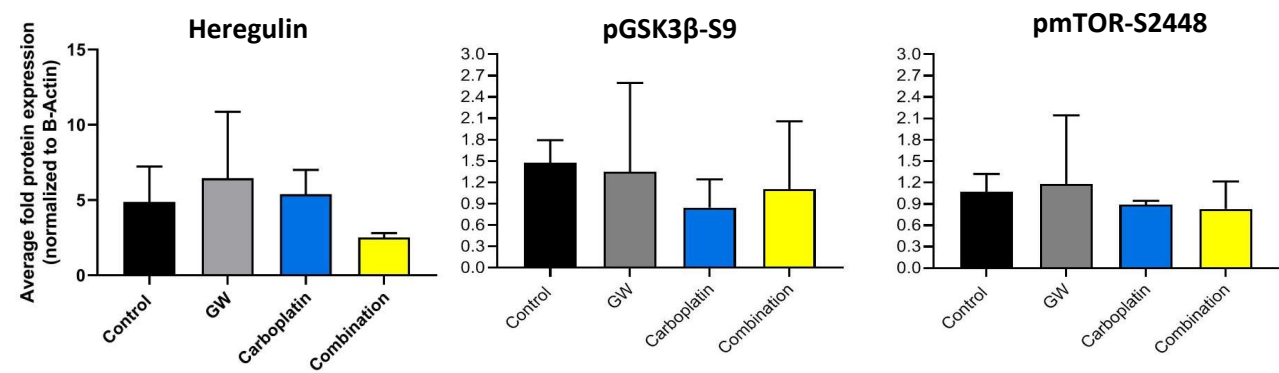

Supplement: Supplementary file 3 — Fig. S3. Protein expression by simple western immunoassay in breast cancer xenograft model MAS98.12 treated with Liver X receptor GW3965 and carboplatin. Average fold protein expression (normalized to β‐actin) of Heregulin, pGSK3β‐S9, and pmTOR‐S2448 as measured by simple western immunoassay for control (n = 3), GW (n = 3), carboplatin (n = 2) and combination (n = 3) treatment groups in MAS98.12 tumors. Data are shown as mean with error bars representing SD. Statistical significance tested by unpaired parametric t‐test. GW: Liver X receptor GW3965. [file MOL2-17-2041-s005.pdf]

Supplementary figure S4

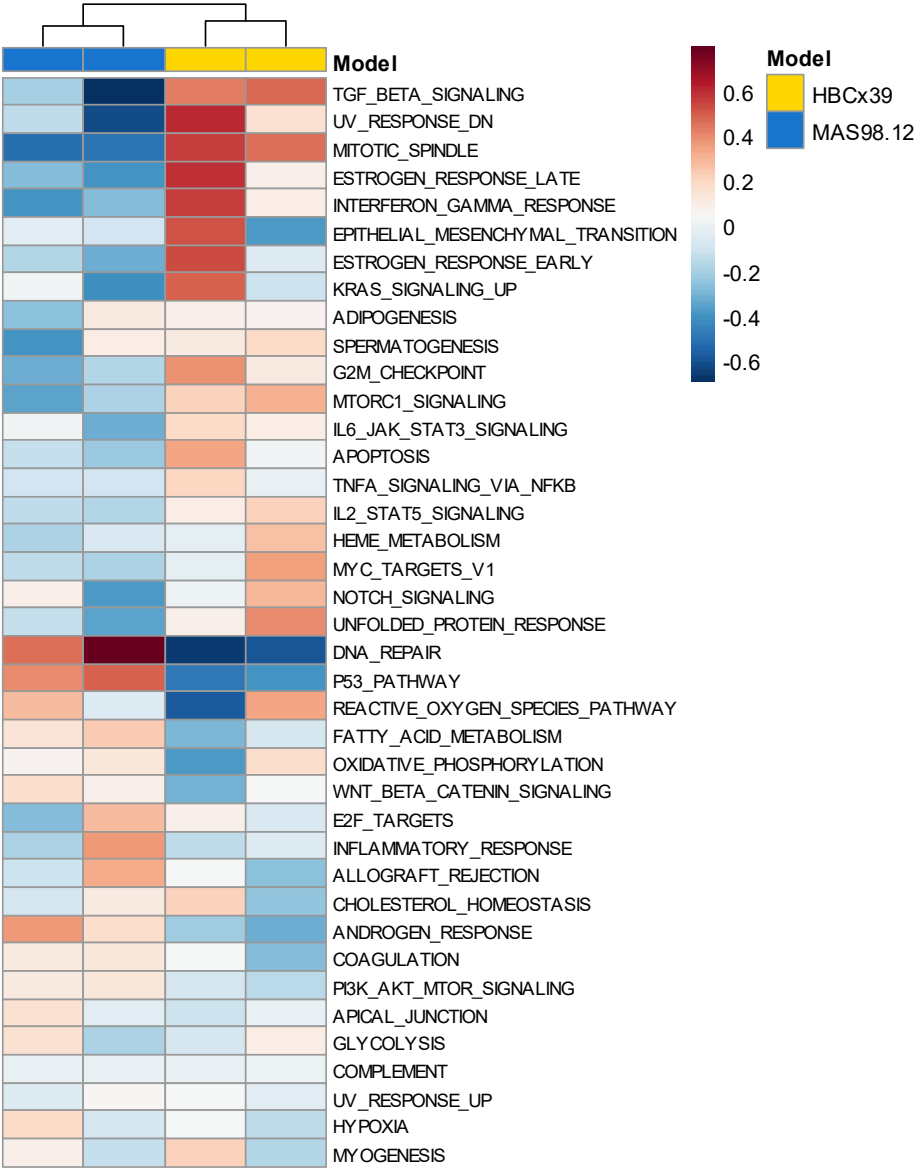

Supplement: Supplementary file 4 — Fig. S4. Heatmap of all Hallmark gene signature scores from Gene Set Variation Analysis (GSVA) in HBCx39 and MAS98.12 control tumors. Hallmark gene signature scores from Gene Set Variation Analysis (GSVA) in HBCx39 (n = 2) and MAS98.12 (n = 2) control tumors with unsupervised clustering of breast cancer xenograft models in heatmap. Scale indicates mean‐centered single sample scores across each pathway. Columns were unsupervised clustered with method complete and distance Euclidean. [file MOL2-17-2041-s001.pdf]

Supplementary figure S5

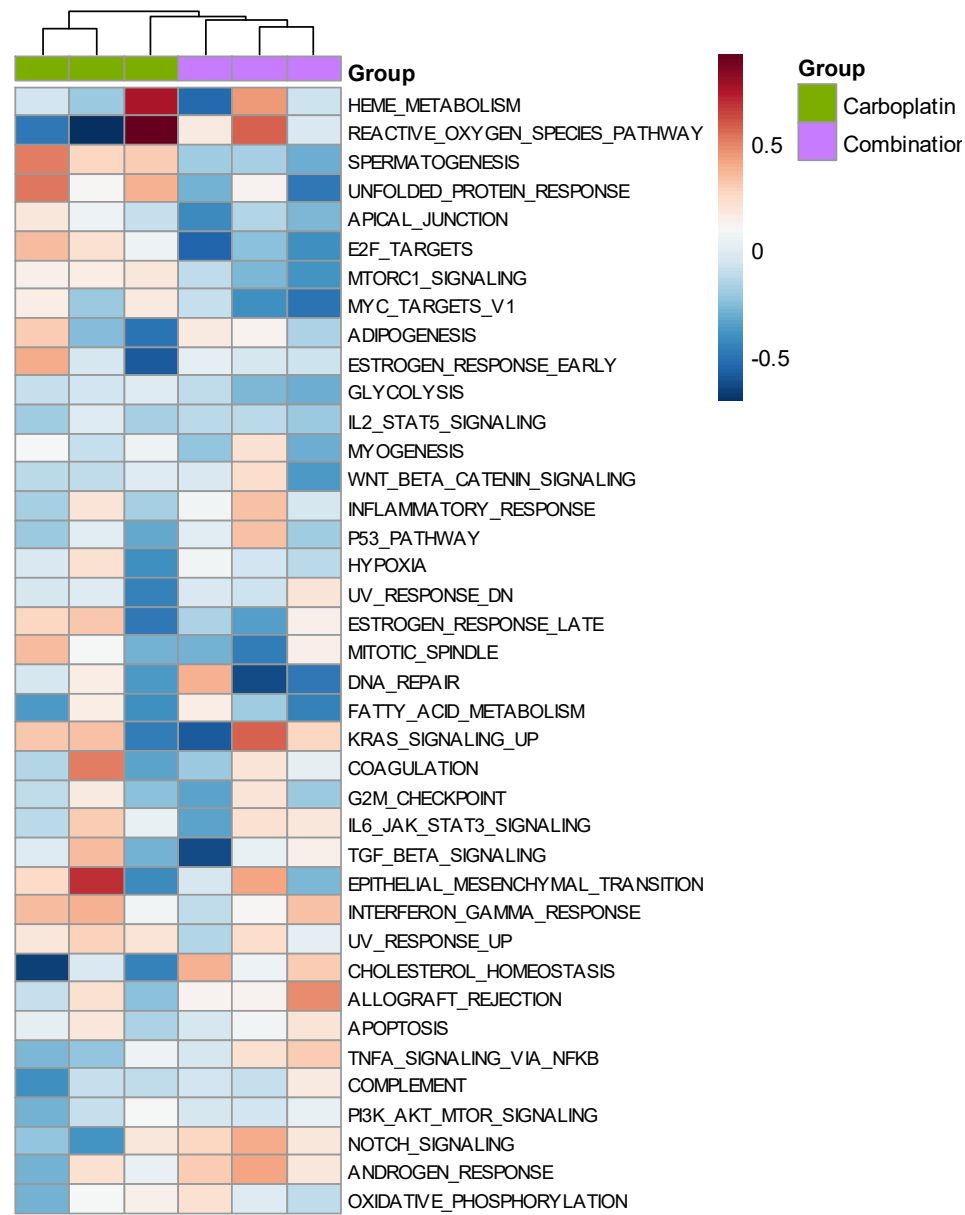

Supplement: Supplementary file 5 — Fig. S5. Heatmap of all Hallmark gene signature scores from Gene Set Variation Analysis (GSVA) in MAS98.12 carboplatin‐ and combination‐treated tumors. Hallmark gene signature scores from Gene Set Variation Analysis (GSVA) in MAS98.12 carboplatin (n = 3) and combination (n = 3) treated tumors with unsupervised clustering of treatment groups in heatmap. Scale indicates mean‐centered single sample scores across each pathway. Columns were unsupervised clustered with method complete and distance Euclidean. [file MOL2-17-2041-s002.pdf]
