# Supplementary material for: Liver X receptors induce antiproliferative effects in basal‐like breast cancer
Source: Mol Oncol. 2023 Jun 30;17(10):2041–55. doi: 10.1002/1878-0261.13476 (PMC10552888; doi:10.1002/1878-0261.13476)
Supplement: Supplementary file 6 — Table S1. Protein expression by reverse‐phase protein array analysis in MAS98.12 and HBCx39 breast cancer xenograft tumors. Difference in expression of 386 proteins in HBCx39 (n = 2) compared with MAS98.12 (n = 2) untreated breast cancer xenograft tumors by reverse‐phase protein array analysis. Significance by Student's t‐test unadjusted and FDR adjusted p‐value. [file MOL2-17-2041-s003.pdf]

Protein	Gene	FC(log2)	p-value	FDR
14-3-3-beta	YWHAB	0.192	0.258	0.501
14-3-3-zeta	YWHAZ	-0.179	0.079	0.327
4E-BP1	EIF4EBP1	-0.162	0.608	0.724
4E-BP1-pS65	EIF4EBP1	-0.367	0.34	0.537
4E-BP1-pT37-T46	EIF4EBP1	-0.24	0.261	0.501
53BP1	TP53BP1	0.541	0.021	0.233
A-Raf	ARAF	0.823	0.016	0.233
A-Raf-pS299	ARAF	-0.297	0.217	0.468
ACC1	ACACA/ACACB	-0.058	0.878	0.919
ACC-pS79	ACACA/ACACB	-0.137	0.745	0.822
AceCS1	ACSS2	0.007	0.985	0.99
ACLY-pS455	ACLY	0.072	0.596	0.715
ACSL1	ACSL1	0.749	0.116	0.375
ACVRL1	ACVRL1	0.112	0.707	0.791
Akt	AKT1/2/3	0.593	0.489	0.642
Akt1	AKT1	-0.06	0.379	0.56
Akt1-pS473	AKT1	-1.354	0.015	0.233
Akt2	AKT2	0.343	0.24	0.491
Akt2-pS474	AKT2	-0.987	0.004	0.21
Akt-pS473	AKT1/2/3	-1.797	0.012	0.233
Akt-pT308	AKT1/2/3	-0.962	0.004	0.21
Ambra1-pS52	AMBRA1	-0.231	0.318	0.535
AMPK-a2-pS345	PRKAA2	-0.114	0.284	0.507
AMPKa	PRKAA1/2	-0.017	0.861	0.908
AMPKa-pT172	PRKAA1/2	0.067	0.736	0.814
AR	AR	0.132	0.418	0.594
ARID1A	ARID1A	0.856	0.108	0.364
ASNS	ASNS	0.373	0.286	0.508
Atg3	ATG3	-0.084	0.369	0.554
Atg4B	ATG4B	0.645	0.043	0.304
Atg5	ATG5	0.025	0.526	0.672
Atg7	ATG7	-0.107	0.479	0.64
ATM	ATM	-0.233	0.325	0.537
ATM-pS1981	ATM	-0.053	0.562	0.691
ATP5H	ATP5PD	0.038	0.826	0.878
ATR	ATR	-0.172	0.204	0.458
ATRX	ATRX	0.359	0.335	0.537
ATR-pS428	ATR	0.009	0.786	0.855
Aurora-A	AURKA	0.411	0.096	0.338
Aurora-ABC-pT288-pT232-pT198	AURKA-C	0.151	0.221	0.473
Aurora-B	AURKB	0.236	0.305	0.526
Axl	AXL	-0.589	0.193	0.446
b-Actin	ACTB	-0.158	0.248	0.498
b-Catenin	CTNNB1	0.151	0.156	0.412
b-Catenin-pT41-S45	CTNNB1	0.491	0.127	0.387

B-Raf	BRAF	0.185	0.342	0.537
B-Raf-pS445	BRAF	-0.131	0.143	0.399
B7-H3	CD276	-0.854	0.036	0.304
B7-H4	VTCN1	0.813	0.075	0.327
Bad-pS112	BAD	-0.195	0.203	0.458
Bak	BAK1	-0.08	0.071	0.327
Bax	BAX	0.043	0.69	0.777
Bcl-xL	BCL2L1	-0.162	0.178	0.429
Bcl2	BCL2	0.563	0.181	0.432
BCL2A1	BCL2A1	-0.082	0.362	0.552
Beclin	BECN1	-0.185	0.161	0.412
Bid	BID	0.011	0.715	0.798
Bim	BCL2L11	0.856	0.08	0.327
BMK1-Erk5-pT218-Y220	MAPK7	0.153	0.205	0.458
BRD4	BRD4	0.323	0.24	0.491
c-Abl	ABL1	-0.29	0.157	0.412
c-Abl-pY412	ABL1	0.12	0.122	0.387
c-IAP2	BIRC3	0.121	0.02	0.233
c-Jun-pS73	JUN	-0.405	0.046	0.304
c-Kit	KIT	2.28	0.009	0.233
c-Met-pY1234-Y1235	MET	0.307	0.081	0.327
c-Myc	MYC	0.162	0.022	0.233
C-Raf	RAF1	0.061	0.27	0.501
C-Raf-pS338	RAF1	-0.327	0.249	0.498
CA9	CA9	-0.7	0.077	0.327
Calnexin	CANX	0.662	0.019	0.233
Caspase-3-cleaved	CASP3	0.076	0.377	0.56
Caspase-7-cleaved-	CASP7	0.104	0.479	0.64
Caveolin-1	CAV1	0.805	0.132	0.394
CD134	TNFRSF4	-0.217	0.223	0.474
CD20	MS4A1	-0.106	0.252	0.5
CD26	DPP4	-0.824	0.328	0.537
CD38	CD38	0.029	0.874	0.919
CD4	CD4	0.014	0.531	0.676
CD44	CD44	-0.128	0.794	0.859
cdc25C	CDC25C	1.12	0.016	0.233
cdc2-pY15	CDK1	0.168	0.169	0.418
Cdc42	CDC42/RAC1	-0.464	0.27	0.501
Cdc6	CDC6	0.39	0.187	0.438
CDK1-pT14	CDK1/2/3	0.646	0.135	0.394
CDKN2A	CDKN2A	-2.195	0.008	0.233
CDT1	CDT1	0.285	0.236	0.486
CHD1L	CHD1L	0.356	0.282	0.507
Chk1-pS296	CHEK1	0.28	0.142	0.399
Chk1-pS345	CHEK1	-0.18	0.42	0.594
Chk2-pT68	CHEK2	-0.533	0.083	0.327
CIITA	CIITA	0.155	0.301	0.526

Claudin-7	CLDN7	0.02	0.954	0.97
COG3	COG3	0.226	0.081	0.327
Collagen-VI	COL6A1	0.306	0.278	0.503
Connexin-43	GJA1	0.304	0.561	0.691
Coup-TFII	NR2F2	-0.17	0.258	0.501
Cox-IV	COX4I1	0.421	0.187	0.438
Cox2	PTGS2	0.398	0.066	0.327
CRABP2	CRABP2	-0.789	0.181	0.432
Creb	CREB1	0.058	0.494	0.644
CREB-pS133	CREB1	0.375	0.189	0.439
CSK	CSK	-0.142	0.41	0.594
CtIP	RBBP8	0.4	0.077	0.327
Cyclin-B1	CCNB1	-0.475	0.186	0.438
Cyclin-D1	CCND1	0.196	0.077	0.327
Cyclin-E1	CCNE1	-0.166	0.196	0.451
D-a-Tubulin	TUBA4A/TUBA3C	-0.311	0.016	0.233
DAPK2	DAPK2	-0.05	0.696	0.781
DDB-1	DDB1	-0.059	0.777	0.852
DDR1	DDR1	0.066	0.609	0.724
DDR1-pY513	DDR1	0.204	0.49	0.642
DJ1	PARK7	0.604	0.062	0.327
DNA-Ligase-IV	LIG4	-0.035	0.781	0.854
DNA-POLG	POLG	-0.416	0.056	0.327
DNMT1	DNMT1	0.231	0.52	0.667
DRP1	DNM1L	0.633	0.037	0.304
DUSP4	DUSP4	-0.088	0.479	0.64
DUSP6	DUSP6	-0.06	0.42	0.594
Dvl3	DVL3	-0.268	0.002	0.21
E-Cadherin	CDH1	0.514	0.45	0.618
E2F1	E2F1	-0.274	0.167	0.415
eEF2	EEF2	0.283	0.062	0.327
eEF2K	EEF2K	0.546	0.03	0.274
EGFR	EGFR	-0.108	0.234	0.486
EGFR-pY1173	EGFR	0.106	0.255	0.501
eIF4E	EIF4E	0.659	0.02	0.233
eIF4E-pS209	EIF4E	0.303	0.133	0.394
eIF4G	EIF4G1	0.623	0.052	0.326
Elk1-pS383	ELK1	0.12	0.33	0.537
Enolase-1	ENO1	1.114	0	0.092
Enolase-2	ENO2	-0.083	0.33	0.537
EphA2	EPHA2	-0.357	0.068	0.327
EphA2-pS897	EPHA2	-0.487	0.043	0.304
EphA2-pY588	EPHA2	-0.305	0.17	0.418
ER-a	ESR1	0.209	0.209	0.46
ER-a-pS118	ESR1	0.165	0.586	0.71
ERCC5	ERCC5	-0.335	0.063	0.327
Erk5	MAPK7	-0.213	0.128	0.387

ERRalpha	ESRRA	0.251	0.366	0.554
Ets-1	ETS1	0.12	0.544	0.684
EVI1	MECOM	-0.191	0.267	0.501
FABP5	FABP5	-0.15	0.313	0.532
FAK	PTK2	-0.098	0.52	0.667
FAK-pY397	PTK2	-0.733	0.134	0.394
FASN	FASN	0.008	0.977	0.985
FGF-basic	FGF2	0.124	0.568	0.694
Fibronectin	FN1	0.706	0.059	0.327
FN14	TNFRSF12A	0.224	0.315	0.533
FOXM1	FOXM1	0.13	0.569	0.694
FOXO3	FOXO3	-0.461	0.069	0.327
FoxO3a-pS318-S321	FOXO3	-0.073	0.454	0.62
FRS2-alpha-pY196	FRS2	-0.164	0.328	0.537
G6PD	G6PD	-0.156	0.271	0.501
Gab2	GAB2	-0.393	0.111	0.368
GATA6	GATA6	0.086	0.409	0.594
GCLC	GCLC	0.207	0.084	0.327
GCLM	GCLM	-0.174	0.277	0.503
GCN5L2	KAT2A	0.103	0.734	0.814
Gli1	GLI1	-0.085	0.418	0.594
Gli3	GLI3	-0.061	0.787	0.855
Glutamate-D1-2	GLUD1	-1.055	0.081	0.327
Glutaminase	GLS	-1.511	0.04	0.304
Granzyme-B	GZMB	-0.129	0.69	0.777
GRB7	GRB7	0.614	0.068	0.327
Grp75	HSPA9	0.522	0.078	0.327
GSK-3a-b-pS21-S9	GSK3A/GSK3B	-0.242	0.163	0.412
GSK-3B	GSK3B	-0.532	0.084	0.327
Gys	GYS1	-0.906	0.025	0.25
Gys-pS641	GYS1	-0.34	0.074	0.327
H2AX-pS139	H2AX	-0.783	0.351	0.547
HER2-pY1248	ERBB2	0.355	0.509	0.658
HER3	ERBB3	0.571	0.021	0.233
HER3-pY1289	ERBB3	0.259	0.021	0.233
Heregulin	NRG1	-2.622	0.079	0.327
HES1	HES1	0.55	0.081	0.327
Hexokinase-I	HK1	-0.195	0.223	0.474
Hexokinase-II	HK2	0.654	0.274	0.503
Hif-1-alpha	HIF1A	-0.073	0.769	0.846
Histone-H3	H3C1-4/6-8/10-12	0.478	0.444	0.612
Histone-H3-pS10	H3C1-4/6-8/10-12	0.131	0.498	0.648
HLA-DQA1	HLA-DQA1	-0.081	0.415	0.594
HLA-DR-DP-DQ-DX	HLA-DRA	0.278	0.1	0.347
HMHA1	ARHGAP45	-0.227	0.66	0.753
HNRNPK	HNRNPK	-0.012	0.823	0.878
HSP27-pS82	HSBP1	0.377	0.002	0.21

HSP60	HSPD1	0.184	0.485	0.642
HSP70	HSPA1A	0.792	0.029	0.274
IDO	IDO1	0.1	0.154	0.41
IGF1R-pY1135-Y1136	IGF1R/INSR	0.102	0.163	0.412
IGFBP2	IGFBP2	0.267	0.437	0.609
IGFRb	IGF1R	0.922	0.058	0.327
IL-6	IL6	0.105	0.171	0.418
INPP4b	INPP4B	-0.398	0.039	0.304
IR-b	INSR	0.975	0.061	0.327
IRF-1	IRF1	0.193	0.356	0.549
IRF-3	IRF3	0.196	0.428	0.6
IRS1	IRS1	0.066	0.153	0.41
IRS2	IRS2	0.124	0.024	0.25
Jagged1	JAG1	-0.086	0.534	0.676
Jak2	JAK2	0.439	0.104	0.358
JNK2	MAPK9	0.138	0.292	0.514
JNK-pT183-Y185	MAPK8	0.188	0.618	0.727
KAP1	TRIM28	0.286	0.04	0.304
LAD1	LAD1	0.018	0.57	0.694
Lasu1	HUWE1	0.191	0.277	0.503
LC3A-B	MAP1LC3A/B	0.029	0.821	0.878
Lck	LCK	-0.02	0.828	0.878
LDHA	LDHA	-0.524	0.056	0.327
LRP6-pS1490	LRP6	-0.012	0.936	0.96
Lyn	LYN	-0.661	0.039	0.304
MAPK-pT202-Y204	MAPK1/MAPK3	-0.786	0.071	0.327
Mcl-1	MCL1	1.021	0.006	0.21
MCT4	SLC16A3	-0.673	0.267	0.501
MDM2-pS166	MDM2	0.299	0.059	0.327
MEK1	MAP2K1	0.164	0.384	0.563
MEK1-p-S217-S221	MAP2K1/MAP2K2	0.179	0.294	0.515
MEK2	MAP2K2	-0.282	0.124	0.387
MelanA	MLANA	0.295	0.053	0.327
Melanoma-gp100	PMEL	0.026	0.876	0.919
MERIT40	BABAM1	0.419	0.045	0.304
MERIT40-pS29	BABAM1	-0.084	0.622	0.729
Merlin	NF2	-0.403	0.233	0.486
MIF	MIF	-0.231	0.164	0.412
MIG6	ERRFI1	-0.097	0.429	0.6
MITF	MITF	0.283	0.087	0.327
Mitofusin-1	MFN1	0.115	0.645	0.738
Mitofusin-2	MFN2	0.748	0.045	0.304
MLKL	MLKL	0.376	0.258	0.501
MMP14	MMP14	-1.012	0.005	0.21
MMP2	MMP2	0.4	0.128	0.387
Mnk1	MKNK1	0.083	0.284	0.507
MRAP	MRAP	0.047	0.63	0.73

MSH2	MSH2	0.855	0.106	0.363
MSH6	MSH6	0.717	0.203	0.458
MSI2	MSI2	-0.251	0.161	0.412
mTOR	MTOR	0.687	0.115	0.375
mTOR-pS2448	MTOR	0.171	0.372	0.554
MYH11	MYH11	0.239	0.217	0.468
Myosin-IIa	MYH9	-0.216	0.322	0.537
Myosin-IIa-pS1943	MYH9	0.401	0.34	0.537
Myt1	PKMYT1	0.042	0.827	0.878
N-Cadherin	CDH2	0.422	0.073	0.327
NAPSIN-A	NAPSA	0.032	0.627	0.729
NDRG1-pT346	NDRG1	-2.373	0.006	0.21
NDUFB4	NDUFB4	-0.243	0.42	0.594
NF-kB-p65-pS536	RELA	0.532	0.068	0.327
Notch1	NOTCH1	-0.095	0.149	0.404
Notch1-cleaved	NOTCH1	0.307	0.146	0.404
Notch3	NOTCH3	-0.131	0.429	0.6
NRF2	NFE2L2	-0.051	0.325	0.537
Oct-4	POU5F1	0.008	0.968	0.978
P-Cadherin	CDH3	0.353	0.053	0.327
p21	CDKN1A	0.027	0.822	0.878
p27-Kip1	CDKN1B	0.002	0.955	0.97
p27-pT157	CDKN1B	0.639	0.306	0.526
p27-pT198	CDKN1B	-0.136	0.59	0.711
p38-MAPK	MAPK11/12/14	-0.066	0.578	0.702
p38-MAPK--pT180-Y182	MAPK11/12/13/14	-0.009	0.968	0.978
p44-42-MAPK	MAPK1/MAPK3	0.194	0.358	0.549
p53	TP53	-2.149	0.029	0.274
p70-S6K1	RPS6KB1	0.374	0.006	0.21
p70-S6K-pT389	RPS6KB1	0.361	0.244	0.495
p90RSK-pT573	RPS6KA1	0.156	0.335	0.537
PAICS	PAICS	-0.15	0.632	0.73
PAK1	PAK1	0.038	0.887	0.921
PAK4	PAK4	-0.198	0.371	0.554
PAR	[PAR Modification]	-0.65	0.473	0.638
PARG	PARG	0.881	0.012	0.233
PARP	PARP1	0.034	0.824	0.878
Patched	PTCH1	0.031	0.842	0.89
PAX6	PAX6	-1.21	0.002	0.21
PAX8	PAX8	-0.821	0.23	0.484
Paxillin	PXN	0.362	0.269	0.501
PD-1	PDCD1	-0.084	0.369	0.554
PD-L1	CD274	0.267	0.113	0.374
Pdcd4	PDCD4	0.125	0.44	0.609
PDHA1	PDHA1	0.018	0.885	0.921
PDHK1	PDK1	-0.073	0.339	0.537
PDK1	PDPK1	-0.481	0.085	0.327

PDK1-pS241	PDPK1	-0.475	0.201	0.458
PEA-15	PEA15	-0.024	0.643	0.738
PEA-15-pS116	PEA15	0.401	0.126	0.387
PERK	EIF2AK3	0.163	0.095	0.338
PHGDH	PHGDH	1.017	0.021	0.233
PHLPP	PHLPP1	0.283	0.009	0.233
PI3K-p110-a	PIK3CA	0.15	0.048	0.311
PI3K-p85	PIK3R1	-0.052	0.623	0.729
PKA-a	PRKAR1A	-1.064	0.018	0.233
PKC-a-b-II-pT638-T641	PRKCA/PRKCB	-0.092	0.467	0.633
PKC-b-II-pS660	PRKCA/B/D/E/H/Q	0.43	0.03	0.274
PKC-delta-pS664	PRKCD	0.177	0.046	0.304
PKCa	PRKCA	0.394	0.164	0.412
PKM2	PKM	0.484	0.006	0.21
PLC-gamma1	PLCG1	-0.144	0.248	0.498
PLC-gamma1-pS1248	PLCG1	-0.026	0.887	0.921
PLC-gamma2-pY759	PLCG2	-0.064	0.44	0.609
PLK1	PLK1	-0.03	0.941	0.963
PMS2	PMS2	-0.89	0.015	0.233
PR	PGR	0.11	0.562	0.691
PRAS40-pT246	AKT1S1	0.161	0.271	0.501
PRC1-pT481	PRC1	0.289	0.136	0.396
PREX1	PREX1	0.53	0.14	0.399
PTEN	PTEN	1.588	0.125	0.387
PTPN12	PTPN12	0.083	0.489	0.642
Puma	BBC3	0.37	0.149	0.404
PYGB	PYGB	-0.333	0.088	0.328
Pyk2-pY402	PTK2B	-0.127	0.594	0.714
Rab11	RAB11A/B	0.591	0.307	0.526
Rab25	RAB25	-0.287	0.341	0.537
Rad23A	RAD23A	-0.25	0.091	0.331
Rad50	RAD50	0.113	0.558	0.691
Rad51	RAD51	-3.284	0.014	0.233
Raptor	RPTOR	-0.189	0.385	0.563
RBM15	RBM15	0.033	0.932	0.96
Rb-pS807-S811	RB1	0.777	0.019	0.233
Rictor	RICTOR	0.318	0.089	0.328
Rictor-pT1135	RICTOR	0.204	0.041	0.304
RIP	RIPK1	-0.387	0.161	0.412
RIP3	RIPK3	-0.06	0.612	0.724
RPA32	RPA2	0.34	0.086	0.327
RPA32-pS4-S8	RPA2	-0.172	0.509	0.658
RRM1	RRM1	-0.079	0.61	0.724
RRM2	RRM2	0.88	0.047	0.304
RSK	RPS6KA1/2/3	0.219	0.333	0.537
RSK1	RPS6KA1	0.167	0.213	0.466
S100A4	S100A4	-0.08	0.109	0.365

S6-pS235-S236	RPS6	0.228	0.625	0.729
S6-pS240-S244	RPS6	0.214	0.641	0.738
SDHA	SDHA	-0.958	0.094	0.338
SFRP1	SFRP1	-2.149	0.029	0.274
SGK1	SGK1	0.055	0.666	0.758
SGK3	SGK3	0.868	0.124	0.387
Shc-pY317	SHC1	0.03	0.689	0.777
SHP-2-pY542	PTPN11	0.146	0.336	0.537
SHP2	PTPN11	-0.119	0.735	0.814
SLC1A5	SLC1A5	0.269	0.559	0.691
Slfn11	SLFN11	0.277	0.268	0.501
Smad1	SMAD1	0.507	0.089	0.328
Smad3	SMAD3	0.135	0.323	0.537
Smad4	SMAD4	0.313	0.304	0.526
SOD2	SOD2	0.086	0.367	0.554
Sox17	SOX17	-0.723	0.348	0.544
Sox2	SOX2	-0.013	0.947	0.967
Src-pY416	SRC	-0.374	0.038	0.304
Src-pY527	SRC	-0.09	0.613	0.724
Stat1-pY701	STAT1	0.253	0.356	0.549
Stat3	STAT3	1.021	0.381	0.561
Stat3-pY705	STAT3	0.638	0.122	0.387
Stat5a	STAT5A	-0.247	0.267	0.501
Stathmin-1	STMN1	0.168	0.016	0.233
STING	STING1	0.245	0.416	0.594
TAZ	WWTR1	0.682	0.147	0.404
TFAM	TFAM	-0.189	0.534	0.676
TFRC	TFRC	1.731	0.015	0.233
TIGAR	TIGAR	2.407	0.536	0.677
TRIM25	TRIM25	0.099	0.455	0.62
TRIP13	TRIP13	-0.002	0.991	0.994
TSC1	TSC1	0.349	0.14	0.399
TTF1	NKX2-1	-0.237	0.794	0.859
Tuberin	TSC2	-0.217	0.215	0.468
Tuberin-pT1462	TSC2	-0.133	0.253	0.5
TUFM	TUFM	0.126	0.313	0.532
Tyro3	TYRO3	-0.063	0.554	0.691
U-Histone-H2B	H2BC3	0.974	0.077	0.327
UBAC1	UBAC1	-0.272	0.059	0.327
ULK1-pS757	ULK1	-0.284	0.141	0.399
UVRAG	UVRAG	-0.053	0.49	0.642
VASP	VASP	0.904	0.062	0.327
VAV1	VAV1	0.734	0.013	0.233
VEGFR-2	KDR	0.713	0.15	0.404
VEGFR-2-pY1175	KDR	-0.119	0.459	0.624
VHL	VHL	0.033	0.55	0.69
Wee1	WEE1	-0.37	0.291	0.514

Wee1-pS642	WEE1	-0.001	0.994	0.994
WIPI1	WIPI1	-0.205	0.357	0.549
WIPI2	WIPI2	-1.19	0.046	0.304
XIAP	XIAP	-0.065	0.687	0.777
XPF	ERCC4	-0.562	0.008	0.233
XRCC1	XRCC1	-0.147	0.485	0.642
YAP	YAP1	0.013	0.919	0.949
YAP-pS127	YAP1	0.245	0.174	0.422
YB1-pS102	YBX1	0.124	0.207	0.46
YES1	YES1	-0.096	0.231	0.484
ZAP-70	ZAP70	-0.024	0.918	0.949
ZEB1	ZEB1	0.311	0.099	0.347
