# Supplementary material for: Liver X receptors induce antiproliferative effects in basal‐like breast cancer
Source: Mol Oncol. 2023 Jun 30;17(10):2041–55. doi: 10.1002/1878-0261.13476 (PMC10552888; doi:10.1002/1878-0261.13476)
Supplement: Supplementary file 7 — Table S2. Protein expression by reverse‐phase protein array analysis in MAS98.12 carboplatin‐ and combination‐treated cancer xenograft tumors. Difference in expression of 386 proteins in the combination (n = 3) compared with carboplatin (n = 3) treated MAS98.12 breast cancer xenograft tumors. Significance by Student's t‐test unadjusted and FDR adjusted p‐value. [file MOL2-17-2041-s007.pdf]

Protein	Gene	FC(log2)	p-value	FDR
14-3-3-beta	YWHAB	0.087	0.404	0.988
14-3-3-zeta	YWHAZ	-0.064	0.47	0.99
4E-BP1	EIF4EBP1	-0.182	0.216	0.988
4E-BP1-pS65	EIF4EBP1	-0.137	0.346	0.988
4E-BP1-pT37-T46	EIF4EBP1	-0.072	0.573	0.992
53BP1	TP53BP1	-0.323	0.12	0.988
A-Raf	ARAF	-0.087	0.624	0.992
A-Raf-pS299	ARAF	0.11	0.6	0.992
ACC1	ACACA/ACACB	0.614	0.086	0.988
ACC-pS79	ACACA/ACACB	0.078	0.841	0.999
AceCS1	ACSS2	0.489	0.027	0.988
ACLY-pS455	ACLY	0.052	0.57	0.992
ACSL1	ACSL1	0.687	0.203	0.988
ACVRL1	ACVRL1	-0.003	0.983	0.999
Akt	AKT1/2/3	-0.838	0.352	0.988
Akt1	AKT1	-0.095	0.541	0.992
Akt1-pS473	AKT1	0.009	0.95	0.999
Akt2	AKT2	0.003	0.985	0.999
Akt2-pS474	AKT2	-0.038	0.807	0.999
Akt-pS473	AKT1/2/3	-0.442	0.095	0.988
Akt-pT308	AKT1/2/3	-0.255	0.179	0.988
Ambra1-pS52	AMBRA1	-0.008	0.958	0.999
AMPK-a2-pS345	PRKAA2	0.069	0.378	0.988
AMPKa	PRKAA1/2	-0.007	0.885	0.999
AMPKa-pT172	PRKAA1/2	0.055	0.809	0.999
AR	AR	0.001	0.989	0.999
ARID1A	ARID1A	-0.355	0.459	0.99
ASNS	ASNS	-0.283	0.184	0.988
Atg3	ATG3	0.041	0.732	0.993
Atg4B	ATG4B	0.044	0.619	0.992
Atg5	ATG5	0.047	0.805	0.999
Atg7	ATG7	0.142	0.49	0.992
ATM	ATM	-0.18	0.347	0.988
ATM-pS1981	ATM	-0.044	0.638	0.992
ATP5H	ATP5PD	0.14	0.409	0.988
ATR	ATR	-0.124	0.327	0.988
ATRX	ATRX	0.024	0.885	0.999
ATR-pS428	ATR	-0.052	0.593	0.992
Aurora-A	AURKA	-0.164	0.242	0.988
Aurora-ABC-pT288-pT232-pT198	AURKA-C	0.08	0.391	0.988
Aurora-B	AURKB	-0.237	0.409	0.988
Axl	AXL	0.242	0.579	0.992
b-Actin	ACTB	0.034	0.745	0.994
b-Catenin	CTNNB1	0.066	0.62	0.992
b-Catenin-pT41-S45	CTNNB1	0.12	0.569	0.992

B-Raf	BRAF	-0.217	0.255	0.988
B-Raf-pS445	BRAF	-0.078	0.313	0.988
B7-H3	CD276	-0.094	0.626	0.992
B7-H4	VTCN1	-0.027	0.761	0.999
Bad-pS112	BAD	0.021	0.619	0.992
Bak	BAK1	0.086	0.096	0.988
Bax	BAX	0.095	0.504	0.992
Bcl-xL	BCL2L1	0.023	0.823	0.999
Bcl2	BCL2	0.053	0.695	0.992
BCL2A1	BCL2A1	0.138	0.021	0.988
Beclin	BECN1	0.092	0.283	0.988
Bid	BID	-0.183	0.117	0.988
Bim	BCL2L11	0.083	0.8	0.999
BMK1-Erk5-pT218-Y220	MAPK7	0.009	0.957	0.999
BRD4	BRD4	-0.148	0.506	0.992
c-Abl	ABL1	-0.053	0.472	0.99
c-Abl-pY412	ABL1	0.194	0.197	0.988
c-IAP2	BIRC3	0.025	0.779	0.999
c-Jun-pS73	JUN	0.25	0.479	0.992
c-Kit	KIT	0.053	0.804	0.999
c-Met-pY1234-Y1235	MET	0.038	0.699	0.992
c-Myc	MYC	0.024	0.872	0.999
C-Raf	RAF1	-0.028	0.887	0.999
C-Raf-pS338	RAF1	0.093	0.564	0.992
CA9	CA9	-0.303	0.398	0.988
Calnexin	CANX	-0.058	0.644	0.992
Caspase-3-cleaved	CASP3	0.105	0.203	0.988
Caspase-7-cleaved-	CASP7	0.196	0.38	0.988
Caveolin-1	CAV1	-0.031	0.887	0.999
CD134	TNFRSF4	0.066	0.607	0.992
CD20	MS4A1	0.011	0.936	0.999
CD26	DPP4	0.072	0.59	0.992
CD38	CD38	0.009	0.934	0.999
CD4	CD4	0.088	0.328	0.988
CD44	CD44	-0.345	0.237	0.988
cdc25C	CDC25C	-0.122	0.499	0.992
cdc2-pY15	CDK1	0.021	0.882	0.999
Cdc42	CDC42/RAC1	0.21	0.188	0.988
Cdc6	CDC6	-0.158	0.079	0.988
CDK1-pT14	CDK1/2/3	-0.069	0.287	0.988
CDKN2A	CDKN2A	-0.063	0.56	0.992
CDT1	CDT1	-0.008	0.938	0.999
CHD1L	CHD1L	-0.01	0.93	0.999
Chk1-pS296	CHEK1	0.463	0.246	0.988
Chk1-pS345	CHEK1	-0.417	0.134	0.988
Chk2-pT68	CHEK2	-0.818	0.083	0.988
CIITA	CIITA	0.007	0.935	0.999

Claudin-7	CLDN7	-0.384	0.307	0.988
COG3	COG3	-0.015	0.886	0.999
Collagen-VI	COL6A1	0.561	0.185	0.988
Connexin-43	GJA1	0.481	0.106	0.988
Coup-TFII	NR2F2	0.015	0.957	0.999
Cox-IV	COX4I1	-0.036	0.941	0.999
Cox2	PTGS2	-0.145	0.1	0.988
CRABP2	CRABP2	0.215	0.328	0.988
Creb	CREB1	0.128	0.25	0.988
CREB-pS133	CREB1	0.034	0.927	0.999
CSK	CSK	0.102	0.615	0.992
CtIP	RBBP8	-0.024	0.785	0.999
Cyclin-B1	CCNB1	-0.252	0.133	0.988
Cyclin-D1	CCND1	0.093	0.306	0.988
Cyclin-E1	CCNE1	0.024	0.852	0.999
D-a-Tubulin	TUBA4A/TUBA3C	0.14	0.306	0.988
DAPK2	DAPK2	0.103	0.406	0.988
DDB-1	DDB1	0.068	0.688	0.992
DDR1	DDR1	-0.066	0.517	0.992
DDR1-pY513	DDR1	0.039	0.801	0.999
DJ1	PARK7	0.164	0.45	0.99
DNA-Ligase-IV	LIG4	0.18	0.041	0.988
DNA-POLG	POLG	0.133	0.21	0.988
DNMT1	DNMT1	-0.323	0.079	0.988
DRP1	DNM1L	0.237	0.294	0.988
DUSP4	DUSP4	0.166	0.176	0.988
DUSP6	DUSP6	-0.267	0.466	0.99
Dvl3	DVL3	0.079	0.496	0.992
E-Cadherin	CDH1	0.075	0.65	0.992
E2F1	E2F1	0.105	0.566	0.992
eEF2	EEF2	0.057	0.69	0.992
eEF2K	EEF2K	-0.059	0.726	0.993
EGFR	EGFR	0.071	0.613	0.992
EGFR-pY1173	EGFR	0.053	0.456	0.99
eIF4E	EIF4E	-0.194	0.369	0.988
eIF4E-pS209	EIF4E	-0.001	0.998	0.999
eIF4G	EIF4G1	-0.221	0.269	0.988
Elk1-pS383	ELK1	-0.035	0.68	0.992
Enolase-1	ENO1	0.014	0.889	0.999
Enolase-2	ENO2	0.046	0.655	0.992
EphA2	EPHA2	-0.006	0.969	0.999
EphA2-pS897	EPHA2	0.19	0.375	0.988
EphA2-pY588	EPHA2	0.091	0.304	0.988
ER-a	ESR1	0.009	0.95	0.999
ER-a-pS118	ESR1	-0.113	0.024	0.988
ERCC5	ERCC5	0.001	0.988	0.999
Erk5	MAPK7	0.012	0.887	0.999

ERRalpha	ESRRA	0.137	0.409	0.988
Ets-1	ETS1	0.077	0.564	0.992
EVI1	MECOM	0.142	0.301	0.988
FABP5	FABP5	0.046	0.664	0.992
FAK	PTK2	0.001	0.992	0.999
FAK-pY397	PTK2	-0.031	0.888	0.999
FASN	FASN	1.089	0.007	0.988
FGF-basic	FGF2	-0.358	0.158	0.988
Fibronectin	FN1	-0.001	0.997	0.999
FN14	TNFRSF12A	-0.222	0.378	0.988
FOXM1	FOXM1	-0.124	0.439	0.99
FOXO3	FOXO3	-0.076	0.707	0.993
FoxO3a-pS318-S321	FOXO3	0.1	0.454	0.99
FRS2-alpha-pY196	FRS2	0.142	0.123	0.988
G6PD	G6PD	0.036	0.74	0.993
Gab2	GAB2	0.262	0.288	0.988
GATA6	GATA6	0.06	0.44	0.99
GCLC	GCLC	-0.038	0.735	0.993
GCLM	GCLM	-0.004	0.981	0.999
GCN5L2	KAT2A	-0.234	0.198	0.988
Gli1	GLI1	0.095	0.427	0.99
Gli3	GLI3	0.237	0.188	0.988
Glutamate-D1-2	GLUD1	0.196	0.368	0.988
Glutaminase	GLS	-0.349	0.217	0.988
Granzyme-B	GZMB	0.246	0.41	0.988
GRB7	GRB7	-0.108	0.625	0.992
Grp75	HSPA9	-0.073	0.346	0.988
GSK-3a-b-pS21-S9	GSK3A/GSK3B	-0.116	0.747	0.994
GSK-3B	GSK3B	-0.033	0.71	0.993
Gys	GYS1	-0.019	0.936	0.999
Gys-pS641	GYS1	-0.081	0.795	0.999
H2AX-pS139	H2AX	-0.318	0.685	0.992
HER2-pY1248	ERBB2	-0.003	0.989	0.999
HER3	ERBB3	0.096	0.488	0.992
HER3-pY1289	ERBB3	-0.009	0.892	0.999
Heregulin	NRG1	-1.009	0.177	0.988
HES1	HES1	0.115	0.399	0.988
Hexokinase-I	HK1	-0.183	0.454	0.99
Hexokinase-II	HK2	-0.326	0.024	0.988
Hif-1-alpha	HIF1A	-0.163	0.6	0.992
Histone-H3	H3C1-4/6-8/10-12	0.31	0.641	0.992
Histone-H3-pS10	H3C1-4/6-8/10-12	0.144	0.113	0.988
HLA-DQA1	HLA-DQA1	0.408	0.35	0.988
HLA-DR-DP-DQ-DX	HLA-DRA	0.041	0.664	0.992
HMHA1	ARHGAP45	0.143	0.428	0.99
HNRNPK	HNRNPK	-0.074	0.456	0.99
HSP27-pS82	HSBP1	0.191	0.573	0.992

HSP60	HSPD1	-0.217	0.214	0.988
HSP70	HSPA1A	-0.064	0.773	0.999
IDO	IDO1	0.053	0.68	0.992
IGF1R-pY1135-Y1136	IGF1R/INSR	0.078	0.522	0.992
IGFBP2	IGFBP2	-0.102	0.303	0.988
IGFRb	IGF1R	-0.013	0.916	0.999
IL-6	IL6	0.087	0.374	0.988
INPP4b	INPP4B	0.175	0.408	0.988
IR-b	INSR	0.426	0.069	0.988
IRF-1	IRF1	-0.149	0.536	0.992
IRF-3	IRF3	0.314	0.343	0.988
IRS1	IRS1	-0.005	0.966	0.999
IRS2	IRS2	0.119	0.449	0.99
Jagged1	JAG1	0.162	0.317	0.988
Jak2	JAK2	0.074	0.764	0.999
JNK2	MAPK9	0.061	0.342	0.988
JNK-pT183-Y185	MAPK8	-0.114	0.085	0.988
KAP1	TRIM28	-0.103	0.43	0.99
LAD1	LAD1	-0.174	0.357	0.988
Lasu1	HUWE1	-0.144	0.305	0.988
LC3A-B	MAP1LC3A/B	0.057	0.685	0.992
Lck	LCK	0.168	0.334	0.988
LDHA	LDHA	-0.051	0.824	0.999
LRP6-pS1490	LRP6	0.059	0.615	0.992
Lyn	LYN	-0.025	0.844	0.999
MAPK-pT202-Y204	MAPK1/MAPK3	0.629	0.206	0.988
Mcl-1	MCL1	0.24	0.122	0.988
MCT4	SLC16A3	-0.372	0.458	0.99
MDM2-pS166	MDM2	-0.019	0.859	0.999
MEK1	MAP2K1	0.01	0.95	0.999
MEK1-p-S217-S221	MAP2K1/MAP2K2	0.009	0.927	0.999
MEK2	MAP2K2	0.076	0.403	0.988
MelanA	MLANA	-0.016	0.888	0.999
Melanoma-gp100	PMEL	0.081	0.387	0.988
MERIT40	BABAM1	-0.028	0.783	0.999
MERIT40-pS29	BABAM1	-0.325	0.08	0.988
Merlin	NF2	-0.296	0.187	0.988
MIF	MIF	-0.158	0.244	0.988
MIG6	ERRFI1	0.161	0.377	0.988
MITF	MITF	0.126	0.467	0.99
Mitofusin-1	MFN1	0.031	0.908	0.999
Mitofusin-2	MFN2	-0.26	0.414	0.988
MLKL	MLKL	-0.029	0.839	0.999
MMP14	MMP14	0.061	0.718	0.993
MMP2	MMP2	-0.115	0.533	0.992
Mnk1	MKNK1	0.049	0.612	0.992
MRAP	MRAP	0.029	0.715	0.993

MSH2	MSH2	-0.155	0.212	0.988
MSH6	MSH6	-0.153	0.29	0.988
MSI2	MSI2	-0.13	0.18	0.988
mTOR	MTOR	-0.021	0.843	0.999
mTOR-pS2448	MTOR	0.038	0.721	0.993
MYH11	MYH11	-0.513	0.124	0.988
Myosin-IIa	MYH9	-0.177	0.337	0.988
Myosin-IIa-pS1943	MYH9	-0.749	0.148	0.988
Myt1	PKMYT1	-0.135	0.158	0.988
N-Cadherin	CDH2	0.03	0.757	0.998
NAPSIN-A	NAPSA	0	0.999	0.999
NDRG1-pT346	NDRG1	-0.096	0.755	0.997
NDUFB4	NDUFB4	0.045	0.719	0.993
NF-kB-p65-pS536	RELA	-0.07	0.633	0.992
Notch1	NOTCH1	0.062	0.751	0.997
Notch1-cleaved	NOTCH1	-0.028	0.805	0.999
Notch3	NOTCH3	0.084	0.61	0.992
NRF2	NFE2L2	0.06	0.172	0.988
Oct-4	POU5F1	0.065	0.613	0.992
P-Cadherin	CDH3	-0.052	0.658	0.992
p21	CDKN1A	0.064	0.166	0.988
p27-Kip1	CDKN1B	-0.046	0.686	0.992
p27-pT157	CDKN1B	-0.237	0.501	0.992
p27-pT198	CDKN1B	-0.085	0.387	0.988
p38-MAPK	MAPK11/12/14	0.198	0.412	0.988
p38-MAPK--pT180-Y182	MAPK11/12/13/14	0.073	0.521	0.992
p44-42-MAPK	MAPK1/MAPK3	0.302	0.26	0.988
p53	TP53	-0.899	0.093	0.988
p70-S6K1	RPS6KB1	0.155	0.26	0.988
p70-S6K-pT389	RPS6KB1	0.159	0.323	0.988
p90RSK-pT573	RPS6KA1	0.051	0.803	0.999
PAICS	PAICS	-0.112	0.181	0.988
PAK1	PAK1	-0.067	0.734	0.993
PAK4	PAK4	-0.006	0.969	0.999
PAR	[PAR Modification]	0.748	0.109	0.988
PARG	PARG	-0.101	0.597	0.992
PARP	PARP1	0.085	0.409	0.988
Patched	PTCH1	0.046	0.737	0.993
PAX6	PAX6	0.156	0.537	0.992
PAX8	PAX8	-0.062	0.768	0.999
Paxillin	PXN	0.211	0.414	0.988
PD-1	PDCD1	0.065	0.384	0.988
PD-L1	CD274	0.345	0.5	0.992
Pdcd4	PDCD4	-0.227	0.346	0.988
PDHA1	PDHA1	0.002	0.991	0.999
PDHK1	PDK1	0.074	0.08	0.988
PDK1	PDPK1	-0.118	0.41	0.988

PDK1-pS241	PDPK1	-0.11	0.28	0.988
PEA-15	PEA15	0.37	0.199	0.988
PEA-15-pS116	PEA15	0.047	0.825	0.999
PERK	EIF2AK3	0.172	0.246	0.988
PHGDH	PHGDH	0.009	0.931	0.999
PHLPP	PHLPP1	-0.313	0.303	0.988
PI3K-p110-a	PIK3CA	0.003	0.978	0.999
PI3K-p85	PIK3R1	0.053	0.673	0.992
PKA-a	PRKAR1A	0.088	0.387	0.988
PKC-a-b-II-pT638-T641	PRKCA/PRKCB	0.192	0.303	0.988
PKC-b-II-pS660	PRKCA/B/D/E/H/Q	0.426	0.35	0.988
PKC-delta-pS664	PRKCD	0.035	0.713	0.993
PKCa	PRKCA	0.402	0.345	0.988
PKM2	PKM	-0.059	0.697	0.992
PLC-gamma1	PLCG1	-0.076	0.329	0.988
PLC-gamma1-pS1248	PLCG1	-0.091	0.566	0.992
PLC-gamma2-pY759	PLCG2	0.148	0.224	0.988
PLK1	PLK1	-0.435	0.23	0.988
PMS2	PMS2	-0.225	0.318	0.988
PR	PGR	-0.001	0.993	0.999
PRAS40-pT246	AKT1S1	-0.182	0.417	0.988
PRC1-pT481	PRC1	-0.005	0.959	0.999
PREX1	PREX1	0.021	0.908	0.999
PTEN	PTEN	0.468	0.417	0.988
PTPN12	PTPN12	-0.139	0.498	0.992
Puma	BBC3	-0.025	0.862	0.999
PYGB	PYGB	0.133	0.265	0.988
Pyk2-pY402	PTK2B	0.069	0.464	0.99
Rab11	RAB11A/B	-0.062	0.778	0.999
Rab25	RAB25	0.188	0.555	0.992
Rad23A	RAD23A	0.123	0.503	0.992
Rad50	RAD50	-0.11	0.643	0.992
Rad51	RAD51	0.443	0.291	0.988
Raptor	RPTOR	-0.015	0.878	0.999
RBM15	RBM15	-0.216	0.51	0.992
Rb-pS807-S811	RB1	-0.05	0.823	0.999
Rictor	RICTOR	-0.209	0.08	0.988
Rictor-pT1135	RICTOR	-0.018	0.887	0.999
RIP	RIPK1	-0.246	0.427	0.99
RIP3	RIPK3	0.044	0.694	0.992
RPA32	RPA2	-0.075	0.655	0.992
RPA32-pS4-S8	RPA2	-0.449	0.105	0.988
RRM1	RRM1	0.036	0.699	0.992
RRM2	RRM2	-0.095	0.293	0.988
RSK	RPS6KA1/2/3	-0.172	0.528	0.992
RSK1	RPS6KA1	-0.095	0.72	0.993
S100A4	S100A4	0.231	0.289	0.988

S6-pS235-S236	RPS6	-0.218	0.665	0.992
S6-pS240-S244	RPS6	-0.169	0.736	0.993
SDHA	SDHA	0.043	0.688	0.992
SFRP1	SFRP1	-0.313	0.267	0.988
SGK1	SGK1	0.289	0.222	0.988
SGK3	SGK3	0.014	0.933	0.999
Shc-pY317	SHC1	-0.047	0.695	0.992
SHP-2-pY542	PTPN11	0.031	0.832	0.999
SHP2	PTPN11	0.288	0.288	0.988
SLC1A5	SLC1A5	-0.075	0.814	0.999
Slfn11	SLFN11	0.104	0.501	0.992
Smad1	SMAD1	-0.129	0.226	0.988
Smad3	SMAD3	0.039	0.697	0.992
Smad4	SMAD4	0.107	0.586	0.992
SOD2	SOD2	-0.194	0.544	0.992
Sox17	SOX17	0.011	0.938	0.999
Sox2	SOX2	0.08	0.397	0.988
Src-pY416	SRC	-0.213	0.632	0.992
Src-pY527	SRC	-0.026	0.855	0.999
Stat1-pY701	STAT1	-0.011	0.87	0.999
Stat3	STAT3	0.099	0.869	0.999
Stat3-pY705	STAT3	-0.333	0.22	0.988
Stat5a	STAT5A	-0.033	0.403	0.988
Stathmin-1	STMN1	0.05	0.542	0.992
STING	STING1	0.208	0.503	0.992
TAZ	WWTR1	-0.065	0.83	0.999
TFAM	TFAM	0.194	0.639	0.992
TFRC	TFRC	-0.434	0.052	0.988
TIGAR	TIGAR	0.734	0.241	0.988
TRIM25	TRIM25	-0.25	0.431	0.99
TRIP13	TRIP13	-0.107	0.299	0.988
TSC1	TSC1	-0.104	0.443	0.99
TTF1	NKX2-1	-1.177	0.285	0.988
Tuberin	TSC2	-0.258	0.246	0.988
Tuberin-pT1462	TSC2	0.077	0.67	0.992
TUFM	TUFM	-0.015	0.848	0.999
Tyro3	TYRO3	-0.007	0.93	0.999
U-Histone-H2B	H2BC3	0.672	0.278	0.988
UBAC1	UBAC1	-0.05	0.625	0.992
ULK1-pS757	ULK1	-0.219	0.329	0.988
UVRAG	UVRAG	0.095	0.377	0.988
VASP	VASP	-0.218	0.191	0.988
VAV1	VAV1	0.54	0.395	0.988
VEGFR-2	KDR	-0.272	0.348	0.988
VEGFR-2-pY1175	KDR	0.051	0.773	0.999
VHL	VHL	-0.095	0.172	0.988
Wee1	WEE1	-0.254	0.244	0.988

Wee1-pS642	WEE1	-0.099	0.407	0.988
WIPI1	WIPI1	-0.301	0.226	0.988
WIPI2	WIPI2	-0.19	0.337	0.988
XIAP	XIAP	-0.063	0.688	0.992
XPF	ERCC4	0.011	0.952	0.999
XRCC1	XRCC1	0.045	0.649	0.992
YAP	YAP1	0.008	0.959	0.999
YAP-pS127	YAP1	0.006	0.971	0.999
YB1-pS102	YBX1	0.001	0.995	0.999
YES1	YES1	0.047	0.741	0.993
ZAP-70	ZAP70	0.676	0.337	0.988
ZEB1	ZEB1	0.158	0.287	0.988
